# Shedding light on the dynamic interplay of positive and negative symptoms of psychosis with Behavioral Tractography

**DOI:** 10.1038/s41746-025-02128-6

**Published:** 2025-11-27

**Authors:** Andrea Imparato, Natacha Reich, Clémence Feller, Laura Ilen, Stephan Eliez, Christopher Graser, Maude Schneider, Corrado Sandini

**Affiliations:** 1 Developmental Imaging and Psychopathology Laboratory, University of Geneva School of medicine, Geneva, Switzerland; 2 Clinical Psychology Unit for Intellectual and Developmental Disabilities, Faculty of Psychology and Educational Sciences, University of Geneva, Geneva, Switzerland; 3 Dana-Farber Cancer Institute, Harvard University, Boston, MA USA

**Keywords:** Diseases, Neurology, Neuroscience, Psychology, Psychology

## Abstract

Our understanding of the interplay between positive and negative psychosis symptoms is constrained by reliance on retrospective assessments, which fail to capture dynamic, short-term symptom-context interactions. Ecological momentary assessment (EMA) offers real-time symptom tracking in naturalistic settings, but its validity and clinical utility remain uncertain. We address this using a recently developed Behavioral-Tractography approach in individuals with 22q11.2 Deletion Syndrome, a high-risk group for psychosis. Through Network-Dimensionality-Reduction, we found that dynamic EMA patterns closely mirror positive/negative symptom structure derived from gold-standard interviews, suggesting shared latent mechanisms across and within individuals and validating EMA’s assessment of dynamic psychosis symptom processes. Behavioral-Tractography provided informative dissection of symptom interactions—particularly between psychotic symptoms and motivational deficits—modulated by psychosis severity and distinguishing predominantly positive vs. negative profiles. Vulnerability to psychological consequences varied independently of average symptom intensity, supporting the added value of EMA and Behavioral-Tractography for complementing clinical assessments and offering a novel lens on psychosis pathophysiology.

## Introduction

Psychosis is a complex psychiatric disorder characterized by multiple symptom dimensions, each with distinct phenomenology and treatment responses, reflecting different underlying pathophysiologies^1^. Still, these symptoms co-occur frequently enough to be considered manifestations of a single disorder^2^. Positive psychotic symptoms, such as delusions and hallucinations, are hallmarks of psychosis which respond effectively to antipsychotic medications targeting dysregulated dopaminergic activity in the mesolimbic pathway^3^. Negative symptoms, on the other hand, can be subdivided into two broad categories: (1) reduced motivation, including avolition and social withdrawal, and (2) diminished emotional and communicative expressiveness^4–6^. The pathophysiology of negative symptoms is less well understood and not entirely overlapping with that of positive symptoms^4,7^, as indicated by their less predictable and often limited response to antipsychotic treatment^8^. A distinction has been proposed between negative symptoms considered secondary consequences of primary positive symptoms (e.g., social isolation stemming from persecutory delusions), which improve with antipsychotic treatment, and primary negative symptoms, which are refractory to such treatments^9^. Such primary negative symptoms may stem from reduced dopaminergic transmission in the mesocortical pathway^10^, potentially accounting for the paradoxical worsening of motivational deficits sometimes associated with high-dose antipsychotic medication^8,9,11^. Distinguishing primary from secondary negative symptoms, however, currently depends largely on retrospectively observed treatment responses, highlighting a critical gap in our ability to tailor interventions effectively^5,9,12,13^.

This lack of knowledge may partially stem from the retrospective nature of psychiatric assessments, which has limited clinical descriptions of psychosis to a temporal resolution of weeks to months^13^. The dynamic relationship between short-term fluctuations in different clinical dimensions and environmental factors occurring over hours or days^14–16^, could however be critical in mediating the pathophysiological interplay between positive and negative symptoms of psychosis^14,17–24^. The recent advent of ambulatory assessment techniques, such as ecological momentary assessment (EMA), has enabled prospective and dynamic measurements of symptoms in the environmental context in which they occur, through questionnaires administered on smartphones multiple times a day, providing a unique window into such dynamic clinical phenomena^16,25^. In this paper, we explore whether a recently developed Behavioral-Tractography approach could help exploit EMA’s full potential in advancing our understanding of the dynamic interplay between different psychosis symptom dimensions.

The first objective of the present study was to investigate whether EMA questionnaires can provide a sufficiently congruent representation of the complex clinical phenomena characterized by gold-standard psychosis assessments^14^. Similarity in psychometric instruments is typically established based on coherent low-dimensional structure, which is considered a reflection of their ability to capture similar underlying latent phenomena^26^. However, traditional dimensionality reduction techniques, which have been utilized to dissect the low-dimensional structure of positive versus negative symptoms^6^, are not directly applicable to dynamically structured EMA data^25,27,28^. It thus remains fundamentally unclear whether similar phenomena are responsible for across-subject variation and within-subject dynamic fluctuations in positive and negative symptom intensity^27,28^. We recently demonstrated that Network Dimensionality Reduction^29^ can characterize the low-dimensional structure of dynamically fluctuating EMA behavioral data, with high consistency across independent samples of typically developing individuals, as well as in youth at high genetic vulnerability for psychosis due to 22q11.2 Deletion Syndrome^30–32^. Specifically, we identified a first rationality dimension that differentiated states of Affective Distress (e.g., *Anxiety, Sadness, Irritation*) from Psychotic-like Experiences (e.g., *Hallucinations, Feeling-Mistrustful, Confusing-Reality-with-Imagination*), which we defined as Cognitive-Distress^32^. A second valence dimension differentiated psychological distress from reversely coded wellbeing states, which were also segregated according to an affective to cognitive dimension, into Lacking-Affective-Wellbeing (e.g., *Lacking-Happiness, Lacking-Relaxation*) and Lacking-Cognitive-Wellbeing (*Lacking-Concentration, Lacking-Motivation, Lacking-Enjoyment*)^32^. Notably similar valence and rationality axes have been observed in studies of mental state attribution in others^33–37^, suggesting that one’s understanding of others’ mental states may be informed by personal experience of psychological fluctuations^38^. Here, we investigated whether the low-dimensional structure of dynamic EMA fluctuations would mirror variation in psychosis phenomenology measured with gold-standard clinical interviews across 22q11DS individuals. Such overlap would suggest similar latent pathophysiology underlying across-subject and within-subject variation in psychosis phenomenology^28^ and represent an important step toward validating EMA measurement of dynamic psychosis symptom fluctuations^14^. Of note, individuals with 22q11DS are characterized by greater severity of negative symptoms and associated cognitive deficits compared to those with idiopathic clinical risk for psychosis^39,40^, yet the correlation structure among symptom dimensions^41^—including the relative dissociation between positive and negative symptoms^42,43^—tightly resembles that of the general population^41,44^. This makes the syndrome uniquely well-suited for testing instruments designed to capture differential expression of positive and negative symptoms.

The second objective of the present study was to shed light on dynamic behavioral pathways linking positive and negative symptoms at short time frames in everyday life. Indeed, beyond demonstrating their validity, the added value of EMA psychosis assessments also critically depends on deriving a sufficiently meaningful characterization of dynamic interactions between symptomatic dimensions and contextual factors, from highly complex time-dependent and multidimensional EMA data^45,46^. In this regard, we recently proposed a novel Behavioral-Tractography analysis pipeline, providing a unique characterization of the broad architecture of behavioral dynamics, combined with a micro-scale characterization of the role of individual EMA behavioral variables^32^. This multi-scale view of behavioral dynamics proved clinically relevant as it differentiated individuals with 22q11DS from those with idiopathic autism spectrum disorder (ASD). In particular, 22q11DS individuals were vulnerable to the development of secondary motivational/cognitive wellbeing deficits, following the experience of social rejection associated with previous psychotic-like cognitive distress. Interestingly, this pathway mirrors the concept of secondary negative symptoms, but at a much faster temporal scale than previously anticipated^47^. Here, we investigated whether Behavioral-Tractography could help dissect behavioral dynamics underlying variation in positive versus negative symptom dimensions, evaluated with gold-standard clinical instruments in individuals with 22q11DS. Given the novelty of this approach, our aim was primarily proof-of-principle: to evaluate whether Behavioral-Tractography can yield additional, clinically relevant insights into how positive and negative symptoms unfold and interact in daily life. By providing a more intuitive characterization of behavioral dynamics, we propose that Behavioral-Tractography could assist in translating insights generated from EMA to improve psychosis clinical assessments.

## Results

### Clinical patterns detected by SIPS dimensionality reduction

**Fig. 1:**
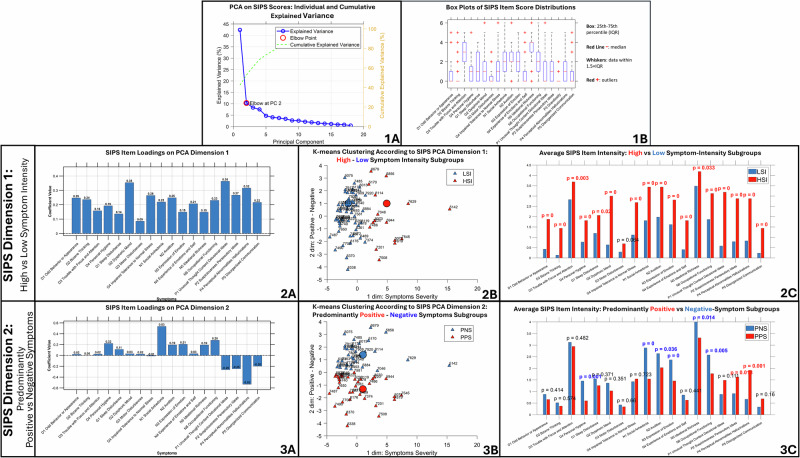
Results of PCA dimensionality reduction on SIPS item scores characterizing main psychosis symptom dimensions. **1A** Cumulative explained variance and elbow point in principal component analysis. The blue line represents the explained variance for each individual principal component (PC), while the green line shows the cumulative explained variance as PCs are successively added. The elbow point suggests an optimal number of PCs to retain. The *x*-axis denotes the number of PCs, and the *y*-axis represents the proportion of variance explained. **1B** Distribution of structured interview for prodromal syndromes (SIPS) item scores. Box plots illustrate score distributions (0–6) across individual items. The *x*-axis represents individual SIPS items, while the *y*-axis shows the score range (0-6). **2A** Bar plot showing the loadings of the first PCA dimension performed on SIPS scores. The *x*-axis represents individual SIPS items, while the *y*-axis displays their corresponding loading values. SIPS-Dimension-1 captured overall symptom severity, as all SIPS items loaded positively along this dimension. **2B** Scatter plot of subject loadings on the first two PC derived from PCA performed on SIPS scores. The *x*-axis represents Dimension-1 (symptom severity), the *y*-axis represents Dimension-2 (positive vs. negative symptoms). Each point represents an individual subject. Color coding reflects cluster membership determined by k-means clustering applied to the first principal component loadings. Blue points indicate subjects with lower overall symptom severity, while red points represent subjects with higher overall symptom severity. **2C** Bar graph comparing average intensity of SIPS items between High-Symptom-Intensity subjects (red) and Low-Symptom-Intensity subjects (blue). The *x*-axis displays individual SIPS items, while the *y*-axis shows the mean score for each item. **3A:** Analogous to (**2A**), but showing loadings on SIPS-Dimension-2, the distinction between positive and negative symptoms, with positive symptoms loading negatively and negative symptoms loading positively. **3B** Analogous to (**2B**), but clustering subjects based on SIPS-Dimension-2 loadings, separating predominantly positive (PPS) from predominantly negative (PNS) symptom subgroups. **3C** Analogous to (**2C**), but comparing mean SIPS item scores between predominantly positive (PPS) and predominantly negative (PNS) symptom subgroups.

Leveraging Principal Component Analysis (PCA) to address the inherent high dimensionality of SIPS data, we extracted two informative principal components (PCs) explaining a substantial portion of the variance (SIPS-Dimension-1: 42.9%, SIPS-Dimension-2: 10.3%) (Fig. 1 Panel-1A).

Dimension 1 captured the propensity for all symptom dimensions to vary as a function of overall disease severity, and manifest as combined clinical presentations in more highly affected individuals. Indeed, every positive, negative, disorganization and general SIPS item loaded positively along this dimension, with strongest contributions for positive symptoms such as *Unusual-Thought-Content, Perceptual-Abnormalities, Persecutory-Ideas* and for the general *Dysphoric-Mood* item (Fig. 1 Panel-2A). K-means clustering on SIPS-Dimension-1 scores separated a Low Symptom Intensity subgroup (LSI; *n* = 53), with lower SIPS-Dimension-1 scores, from a High Symptom Intensity subgroup (HSI; *n* = 15) (Fig. 1 Panels-2B). Compared to LSI the HSI presented higher intensity of all SIPS items except for *Motor-Disturbances* (Fig. 1 Panels-2C).

When controlling for overall disease severity, SIPS-Dimension-2 captured an additional tendency for individuals to present with either a predominance of positive symptoms, which loaded positively, or negative symptoms, which loaded negatively (Fig. 1 Panels-3A). K-means clustering on PC2 scores separated a first Predominantly Positive Symptoms subgroup (PPS; *n* = 31) with higher SIPS-Dimension-2 scores, displaying significantly higher intensity for some positive symptoms (*Hallucinations and Persecutory-Ideas*), from a second Predominantly Negative Symptoms subgroup (PNS; *n* = 37), with lower SIPS-Dimension-2 scores, characterized by higher intensity in all negative symptoms except for *Experience-of-Emotions* as well as higher *Impaired-Personal-Hygiene* disorganization item (Fig. 1 Panels-3B-C).

There were no significant differences in demographic information (age and gender) between subjects within either cluster pair (HSI vs. LSI and PPS vs. PNS; See Table 1 for details). Rates of Psychotic Disorders were higher in the HSI subgroup compared to the LSI subgroup (LSI = 0%, HSI = 25%, *p* < 0.001), but did not differ between the PPS and PNS subgroups (PPS = 6.06%, PNS = 5.71%, *p* = 0.95). Rates of other psychiatric comorbidities associated with 22q11DS^31^—including ADHD, anxiety disorders, and mood disorders—did not differ significantly across subgroups (see Table 1 for details). Full-Scale IQ was higher in LSI compared to HSI (LSI = 75,65 ± 13,07, HSI = 65, 40 ± 13,45, *p* = 0.01) while it did not differ across PPS and PNS subgroups (PPS = 73,22 ± 12,17, PNS = 73,19 ± 15,46, *p* = 0.99).

**Table 1:** Demographic and clinical (KSDAS) information for the four populations

	Cluster 1		T-stat/Chi2	*P* value	Cluster 2		T-stat/Chi2	*P* value
	1	2			1	2		
Age	19.48 (5.17)	18.88 (5.36)	0.40	0.69	20.09 (5.37)	18.62 (4.97)	−1.18	0.24
Gender: M/F	27/25	10/6	0.55	0.46	21/12	16/19	2.20	0.14
Assessments EMA	25.62(11.23)	25.06 (9.92)	0.18	0.86	26.33 (11.53)	24.69 (10.3)	-0.62	0.54
Full scale IQ	75.65 (13.07)	65.40 (13.45)	2.6	0.01	73,19 (15.46)	73.22 (12.17)	0.01	0.99
ADHD	30 (57.7%)	11 (68.75%)	0.62	0.42	20 (57.14%)	21 (63.64%)	0.3	0.58
Anxiety disorders	21 (40.4%)	10 (62.5%)	2.41	0.12	15 (42.86%)	16 (48.48%)	0.22	0.64
Depressive disorders	5 (9.6%)	4 (25%)	2.5	0.11	3 (8.57%)	6 (18.18%)	1.37	0.24
Psychotic disorders	0	4 (25%)	13.81	2.02E-04	2 (5.71%)	2 (6.06%)	0.004	0.95
Conduct disorders	0	1 (6.25%)	3.3	0.07	0	1 (3.03%)	1.07	0.3
Oppositional defiant disorders	0	1 (6.25%)	3.3	0.7	0	1 (3.03%)	1.07	0.3
Bipolar disorder	0	1 (6.25%)	3.3	0.7	0	1 (3.03%)	1.07	0.3
Obsessive compulsive disorders	0	1 (6.25%)	3.3	0.7	1 (2.86%)	0	0.96	0.33
PTSD	0	1 (6.25%)	3.3	0.7	1 (2.86%)	0	0.96	0.33

Gender and KSADS symptom group differences were tested using Chi-Square tests. Continuous variables were tested using Two-Sample T-Tests. Full scale IQ differed significantly between LSI and HSI subgroups.

This two-dimensional framework based on PCA-derived SIPS dimensions, which were largely consistent with previous reports in 22q11DS^42,43^ and in the general population^41,44^, provided a basis to investigate the relationships between SIPS symptom patterns and EMA variables.

### Correlation between SIPS clinical pattern and EMA symptom intensity

SIPS-Dimension-1, reflecting overall psychotic symptom severity, demonstrated strong positive correlations with the average intensity of all EMA items related to positive psychotic symptoms, such as *Hallucinations* (*R* = −0.49, *p* < 0.001), *Feeling-Unsafe* (*R* = −0.57, *p* < 0.001), and *Confusing-Reality-with-Imagination* (*R* = -0.42, *p* < 0.001) and a significant while weaker associations with all affective distress variables such as *Sadness* (*R* = −0.35, *p* < 0.001), *Irritation* (*R* = −0.33, *p* < 0.01), *Anxiety* (*R* = −0.26, *p* < 0.03), *Feeling-Rejected* (*R* = −0.41, *p* < 0.001) (Table 2). Indeed, the average intensity of all such affective and cognitive distress variables, as well as *Fatigue, Lacking-Physical-Activity and Lacking-Confidence* (all *p* < 0.001) were significantly increased in the HSI compared to LSI, who instead reported higher *Lacking-Concentration* (*p* < 0.001), *Lacking-Motivation* (*p* < 0.001), *Lacking-Enjoyment* (*p* < 0.001) and *Finding-Activity-Difficult* (*p* < 0.02) (Table 2).

**Table 2:** Symptoms intensities measured via ecological momentary assessment (EMA) across populations and their correlations with psychopathology dimensions derived from SIPS-PCA

	High-/low-symptom-intensity dimension							Predominant positive–negative symptom Dimension						
EMA symptoms	Mean intensity LSI	Std intensity LSI	Mean intensity HSI	Std intensity HSI	*p* value HSI-LSI	*R*	*p* value	Mean intensity PNS	Std intensity PNS	Mean intensity PPS	Std intensity PPS	*p* value PPS-PNS	*R*	*p* value
Lack relaxation	3.26	1.55	3.22	1.72	0.71	0.00	0.98	3.40	1.57	3.10	1.60	0.00	0.12	0.3
Loneliness	1.62	1.30	1.84	1.39	0.00	0.28	0.02	1.69	1.34	1.64	1.31	0.44	−0.1	0.3
Anxiety	1.81	1.41	2.09	1.49	0.00	0.26	0.03	1.90	1.45	1.85	1.42	0.53	−0.0	0.5
Lack happiness	3.38	1.63	3.47	1.82	0.36	0.08	0.51	3.57	1.66	3.23	1.68	0.00	0.11	0.3
Irritation	1.45	1.12	1.84	1.48	0.00	0.33	0.01	1.51	1.21	1.57	1.24	0.27	−0.1	0.1
Sensory issue	1.23	0.78	1.80	1.25	0.00	0.53	0.00	1.39	1.02	1.34	0.86	0.30	−0.0	0.8
Lack excitement	5.28	1.94	5.30	2.09	0.89	−0.16	0.19	5.29	1.97	5.28	1.99	0.90	0.10	0.4
Sadness	1.51	1.22	2.06	1.62	0.00	0.35	0.00	1.49	1.18	1.78	1.47	0.00	−0.2	0.0
Lack confidence	3.56	1.73	4.01	1.62	0.00	0.07	0.59	3.79	1.76	3.53	1.66	0.00	0.08	0.5
Feeling rejected	1.32	0.92	1.99	1.56	0.00	0.41	0.00	1.47	1.12	1.47	1.14	0.99	−0.1	0.2
Feeling unsafe	1.29	0.94	2.40	1.91	0.00	0.57	0.00	1.45	1.16	1.65	1.46	0.00	−0.1	0.3
Confusion	1.18	0.75	1.52	1.17	0.00	0.42	0.00	1.33	1.05	1.19	0.65	0.00	0.00	0.9
Hallucinations	1.21	0.81	2.18	1.75	0.00	0.49	0.00	1.33	1.05	1.53	1.28	0.00	−0.1	0.3
Feeling tired	3.09	1.85	3.63	1.56	0.00	0.23	0.06	3.06	1.83	3.37	1.77	0.00	0.00	1.0
Lack motivation	4.41	1.76	4.03	2.07	0.00	−0.15	0.23	4.45	1.74	4.20	1.93	0.00	0.10	0.4
Lack physical activity	4.77	1.95	5.16	1.75	0.00	−0.06	0.60	4.94	1.88	4.78	1.94	0.08	0.04	0.7
Finding activity difficult	1.72	1.37	1.55	1.12	0.02	0.18	0.14	1.75	1.37	1.61	1.25	0.04	0.18	0.1
Lack enjoing actvity	3.23	1.62	2.77	1.75	0.00	−0.21	0.09	3.14	1.53	3.11	1.78	0.70	−0.0	0.9
Lack concentration	4.10	1.94	3.69	2.07	0.00	−0.14	0.25	4.07	2.01	3.94	1.94	0.17	0.07	0.5
Being alone	0.36	0.48	0.35	0.48	0.54	0.03	0.84	0.38	0.49	0.33	0.47	0.03	−0.0	0.8

Mean intensity (LSI, HSI, PNS, PPS) and Std intensity (LSI, HSI, PNS, PPS) report the average and standard deviation of EMA symptom intensity scores for Low Symptom Intensity (LSI), High Symptom Intensity (HSI), Predominant Negative Symptoms (PNS), and Predominant Positive Symptoms (PPS) populations. *p* value (HSI-LSI, PPS-PNS) displays the results of independent two-sample t-tests comparing mean symptom intensities between LSI and HSI populations, as well as between PPS and PNS subgroups. Columns R and *p* value represent the Pearson correlation coefficients and significance levels for the relationships with SIPS-PCA Dimension 1 (High-Low Intensity) and Dimension 2 (Positive-Negative Symptoms).

**Fig. 2:**
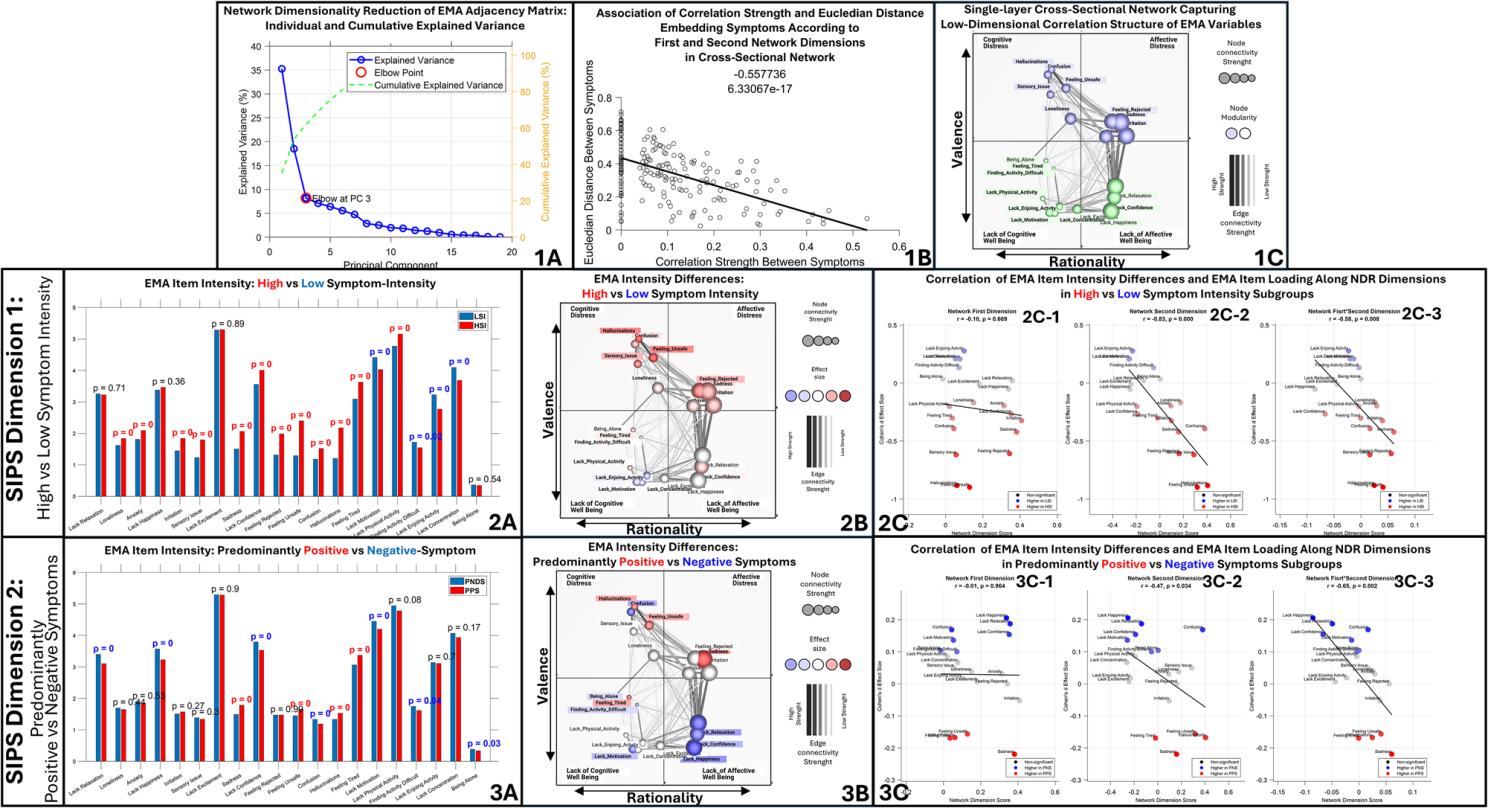
EMA Low-dimensional network structure and its relation to SIPS symptom dimensions. **1A** Principal component analysis (PCA) of the 2D-network adjacency matrix (capturing association strength between EMA variable fluctuations, after adjusting for subject-level intensity via mixed-effects regression). The blue line shows explained variance for each principal component (PC); the green line shows cumulative explained variance. The elbow point indicates the optimal number of PCs. Axes: *x* = number of PCs, *y* = variance explained (0–1). **1B** Association between Euclidean distance (from NDR) and empirically observed correlations between EMA variables, estimating accuracy of the NDR representation. **1C** Cross-sectional EMA co-occurrence network in the 22q11DS sample. Nodes represent psychological/contextual variables; edges represent cross-sectional associations (thickness/transparency = strength). Node size = summed connectivity. Node position reflects two NDR-derived dimensions: horizontal (Rationality): cognitive (left) vs affective (right). Vertical (Valence): psychological distress (top) vs lack of wellbeing (bottom). **2A** Bar graph of average EMA item intensity in High-Symptom-Intensity (HSI, red) vs Low-Symptom-Intensity (LSI, blue) groups, defined by SIPS-Dimension-1. Axes: *x* = EMA items, *y* = mean score. **2B** Cross-sectional EMA co-occurrence network (as in **1C**). Node color scales with effect size of intensity differences between HSI and LSI. **2C** Correlation of EMA intensity effect sizes (Cohen’s d: HSI vs LSI) with NDR coordinates. Points = EMA items, colored by effect direction (red = higher in HSI; blue = higher in LSI; black = ns). Regression lines show relationships with NDR dimensions. **2C1** *x* = Rationality dimension (cognitive–affective). **2C2** *x* = Valence dimension (lack of wellbeing–distress). **2C3** *x* = product of first two coordinates. *y*-axis (all): Cohen’s *d*. **3A**. Analogous to (**2A**), but comparing Predominantly-positive (PPS, red) vs Predominantly-negative (PNS, blue) groups (SIPS-Dimension-2). **3B** Analogous to (**2B**), with node color reflecting PPS vs PNS intensity differences. **3C** Analogous to (**2C**), correlating EMA intensity differences (PPS vs PNS) with NDR coordinates.

SIPS-Dimension-2, distinguishing negative and positive symptom dimensions, was negatively correlated with EMA *Sadness* (*R* = -0.23, *p* < 0.001). *Sadness* was indeed significantly increased in the PPS subgroup, who also reported higher *Hallucinations, Feeling-Unsafe* and *Fatigue* (all *p* < 0.001) compared to the PNS subgroup (Table 2). The PNS subgroup instead reported significantly higher intensity for most EMA items reflecting lack of well-being, including *Lacking-Relaxation, Lacking-Happiness, Lacking-Confidence, Lacking-Motivation* (all *p* < 0.001) as well as higher *Confusing-Reality-with-Imagination* (*p* < 0.001) and higher social isolation (*Being-Alone, p* = 0.03) compared to the PPS group (Table 2 and Fig. 2 Panel-3A). Overall, this suggests that SIPS-Dimesion-2 differences of negative vs positive symptom dimensions across PNS and PPS subgroups were mirrored by similar differences in average intensity of EMA variables measuring psychological distress vs lacking psychological wellbeing.

### Low-dimensional structure of dynamic EMA fluctuations and correspondence with SIPS dimension

In accordance with previous findings^32^, only the first 2 dimensions derived from Network Dimensionality Reduction (NDR), accounting for 35% and 18% of variance in the network structure, meaningfully characterized the propensity for EMA variables to co-occur, as estimated by a significant negative correlation between Euclidian distance separating variables, and empirically observed association strength (*R* = −0.55, *p* < 0.001) (Fig. 2 Panel-1B). Consistent with previous literature^32^, the first NDR Dimension differentiated affective from cognitive variables along a right-to-left horizontal *Rationality* axis, while the second dimension differentiated psychological distress from lacking psychological wellbeing along an upper-to-lower vertical valence axis. Embedding EMA variables according to such dimensions, hence resulted in four network quadrants (Fig. 2 Panel-1C):

- Upper-right: Affective-Distress (*Sadness, Irritation, Anxiety, Feeling-Rejected*).
- Lower-right: Lacking-Affective-Well-Being (*Lacking-Happiness, Lacking-Relaxation, Lacking-Confidence*).
- Lower-left: Lacking-Cognitive-Wellbeing (*Lacking-Motivation, Lacking-Concentration, Lacking-Enjoyment*).
- Upper-left: Cognitive/Psychotic-Distress (*Hallucinations*, *Feeling-Unsafe, Confusing-Reality-with-Imagination)*.

*Loneliness* had an intermediate position in the upper portion of the network, linking social isolation (*Being-Alone*) in the lower-left Lacking-Cognitive-Wellbeing quadrant to upper-left Cognitive-Distress and upper-right Affective-Distress variables.

Interestingly, NDR Dimensions capturing the propensity for EMA variables to fluctuate together also tightly recapitulated the pattern of correlation between the average intensity of EMA variables and SIPS dimensions. Specifically, the strength of SIPS-Dimensions-1-EMA association was significantly anti-correlated with loading of EMA-items along the NDR valence dimension (*R* = −0.83, *p* < 0.001) (Fig. 2 Panel-2C-2). As such EMA items reported with higher intensity in the HSI compared to the LSI subgroup, preferentially clustered in the upper psychological distress portion of the EMA-Network, with particularly strong differences for Psychotic/Cognitive-Distress items clustering in the upper-left network quadrant (Fig. 2 Panel-2B).

SIPS-Dimension-2, capturing the differential Negative-vs-Positive symptom severity, also showed associations with EMA-NDR dimensions, which were particularly strong for the product of Valence and Rationality dimensions (*R* = 0.65, *p* < 0.001) (Fig. 2 Panel-3C-3). As such, EMA items that were increased in the PPS subgroup, clustered preferentially in the upper-right Affective-Distress EMA quadrant, including in particular *Sadness*, as well as *Hallucinations, Feeling-Unsafe and Feeling-Tired*, clustering in the upper-right portions of their respective Cognitive-Distress and Lacking-Cognitive-Wellbeing quadrants. EMA items that were increased in PNS individuals instead clustered preferentially in the lower-left Lacking-Cognitive-Wellbeing variables (*Lacking-Motivation, Being-Alone, Finding-Activity-Difficult*) and lower-right Lacking-Affective-Wellbeing variables (*Lacking-Happiness, Lacking-Relaxation, Lacking-Confidence*). The only psychological distress variable to be increased in the PNS subgroup was *Confusing-Reality-with-Imagination*, located in the leftmost portion of the Cognitive-Distress quadrant (Fig. 2 Panel-3B).

Such correspondence in low-dimensional structure would suggest that latent phenomena underpinning variation in SIPS positive vs negative symptom intensity across subjects may be linked to mechanisms underpinning short term dynamic fluctuation of EMA symptom intensity, reported by individual participants, from one assessment to the next. This would support the validity of EMA for measuring fluctuations in the same positive and negative symptom dimensions that are measured retrospectively with current gold-standard clinical instruments. We, therefore, next explored whether EMA could provide additional insights on dynamic interplay between symptom dimensions in the flow of daily life.

### Association between SIPS-PCA dimension and EMA dynamic network structure

**Fig. 3:**
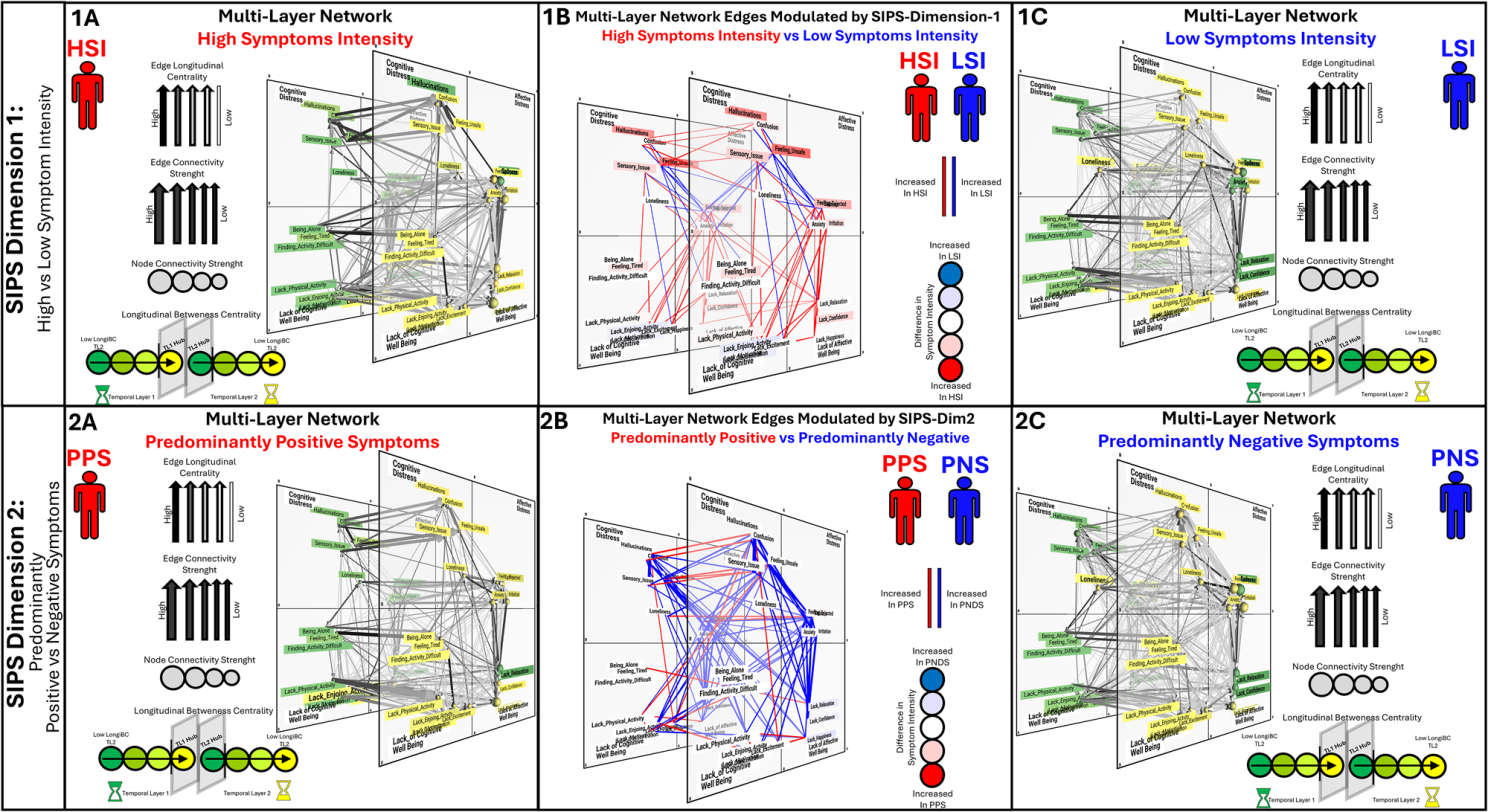
EMA temporal network architecture and symptom-related modulation of behavioral dynamics. Figures were generated using dedicated open-source software (https://dev.mlnetwork-diplab.ch/; https://github.com/andreaimparato/Behavioral-Tractography-Toolbox/tree/main/MLNetwork). For each figure, a link to an online platform provides an interactive 3D visualization. **1A, 1C, 2A, 2C** depict Multi-Layer Temporal Networks (MLTNs) constructed separately for four populations: High-Symptom-Intensity (**1A**), Low-Symptom-Intensity (**1C**), Predominantly-Positive-Symptom (**2A**), and Predominantly-Negative-Symptom (**2C**). The 3D multilayer structure represents behavioral dynamics. Cross-sectional connections appear within temporal layers, while longitudinal edges extend along the *Z*-axis, linking variables from Temporal Layer 1 (TL-1, left) to Temporal Layer 2 (TL-2, right). Node placement within each layer follows Network Dimensionality Reduction, grouping variables that are closely related cross-sectionally. The slope of longitudinal edges reflects the likelihood of dynamic interactions across temporal assessments. Edge thickness indicates the strength of associations from mixed-model regression, while arrows denote edges belonging to shortest paths connecting variables across layers. Edge transparency reflects the number of shortest paths traversing an edge. Node color encodes longitudinal betweenness centrality. A reverse-coded green-to-yellow gradient across TL-1 and TL-2 highlights temporal flow: TL-1 variables shade from green to yellow according to their propensity to act as gateways toward future states, while TL-2 variables shade from yellow to green to represent their role as funnels from past states. Variables acting as longitudinal hubs are highlighted in bold. Node size indicates summed connectivity strength. **1B**, **2B** show MLTNs modulated by SIPS dimensions. Edges are color-coded to indicate effects of SIPS dimensions on association strength between EMA variables, while node color reflects their influence on average intensity. **1B** represents SIPS-Dimension 1 (overall symptom intensity): red edges and nodes denote stronger associations or higher intensity in High-Symptom-Intensity, while blue indicates stronger associations or higher intensity in Low-Symptom-Intensity. **2B** represents SIPS-Dimension 2 (Predominantly-Positive vs. Predominantly-Negative Symptoms): red denotes stronger associations or higher intensity in Predominantly-Positive Symptoms, and blue in Predominantly-Negative Symptoms. Links to 3D interactive visualization: https://dev.mlnetwork-diplab.ch/3dvisualizer/net_dim1_hsi/. https://dev.mlnetwork-diplab.ch/3dvisualizer/net_dim1_lsi/. https://dev.mlnetwork-diplab.ch/3dvisualizer/net_dim2_pns/. https://dev.mlnetwork-diplab.ch/3dvisualizer/net2_dim2_pps/. https://dev.mlnetwork-diplab.ch/3dvisualizer/dim2_pns_pps/. https://dev.mlnetwork-diplab.ch/3dvisualizer/dim1_lsi_hsi/.

We firstly investigated how SIPS-PCA dimensions modulated the strength of individual associations between pairs of EMA variables. In the 3D-MLN view, cross-sectional connections that were significantly modulated by SIPS-PCA dimensions were represented in cross-sectional temporal layers, while time-lagged connections connected temporal layers along the left-to-right temporal axis (Fig. 3).

SIPS-Dimension-1, capturing overall symptom severity, was associated with weaker cross-sectional connections between Psychotic/Cognitive-Distress and Affective-Distress EMA items, while cross-sectional connections among Psychotic/Cognitive-Distress items (*Confusing-Reality-with-Imagination* and *Feeling-Unsafe)* were stronger. EMA Psychotic/Cognitive-Distress symptoms were furthermore more strongly predictive of each other across time and somewhat less tightly predicted by previous Affective-Distress. Affective-Distress symptoms were instead more strongly associated with both concomitant and future lack of well-being states (Fig. 3 Panel-1B).

SIPS-Dimension-2 modulated several of the same alterations in EMA-network structure as SIPS-Dimension-1, capturing the relative contribution of predominantly-positive vs predominantly-negative symptoms to such alterations. In particular, SIPS-Dimension-2 modulated similar strengthening of cross-sectional and longitudinal connections between Psychotic/Cognitive-Distress items, as well as the dissociation of these items from Affective-Distress, demonstrating that such alterations were associated with the presence of Predominantly-Positive-Symptom (PPS) patterns (Fig. 3 Panel-2B). This would suggest that in individuals with more severe SIPS positive psychotic symptoms, who clustered in HSI and PPS subgroups, EMA Psychotic/Cognitive-Distress experiences were not only more intense, but also fluctuated as more coherent and temporally stable events, that were less tightly linked to other forms of psychological distress.

SIPS-Dimension-2 also modulated the strengthening of connections between Affective-Distress and Lacking-Affective-Wellbeing variables observed for SIPS-Dimension-1, demonstrating that such alterations were specifically driven by individuals with PNS. Moreover, in PNS individuals, Lacking-Affective-Wellbeing variables were also more tightly predicted by previous Affective-Distress. This would suggest that the severity of SIPS negative symptoms was reflected both in the average intensity of EMA psychological well-being states and in the dynamic phenomenology of such states, which were more tightly associated with concomitant and previous Affective-Distress.

### Dissecting psychological contextual dynamics linked to positive-negative dimensions with Behavioral Tractography

**Fig. 4:**
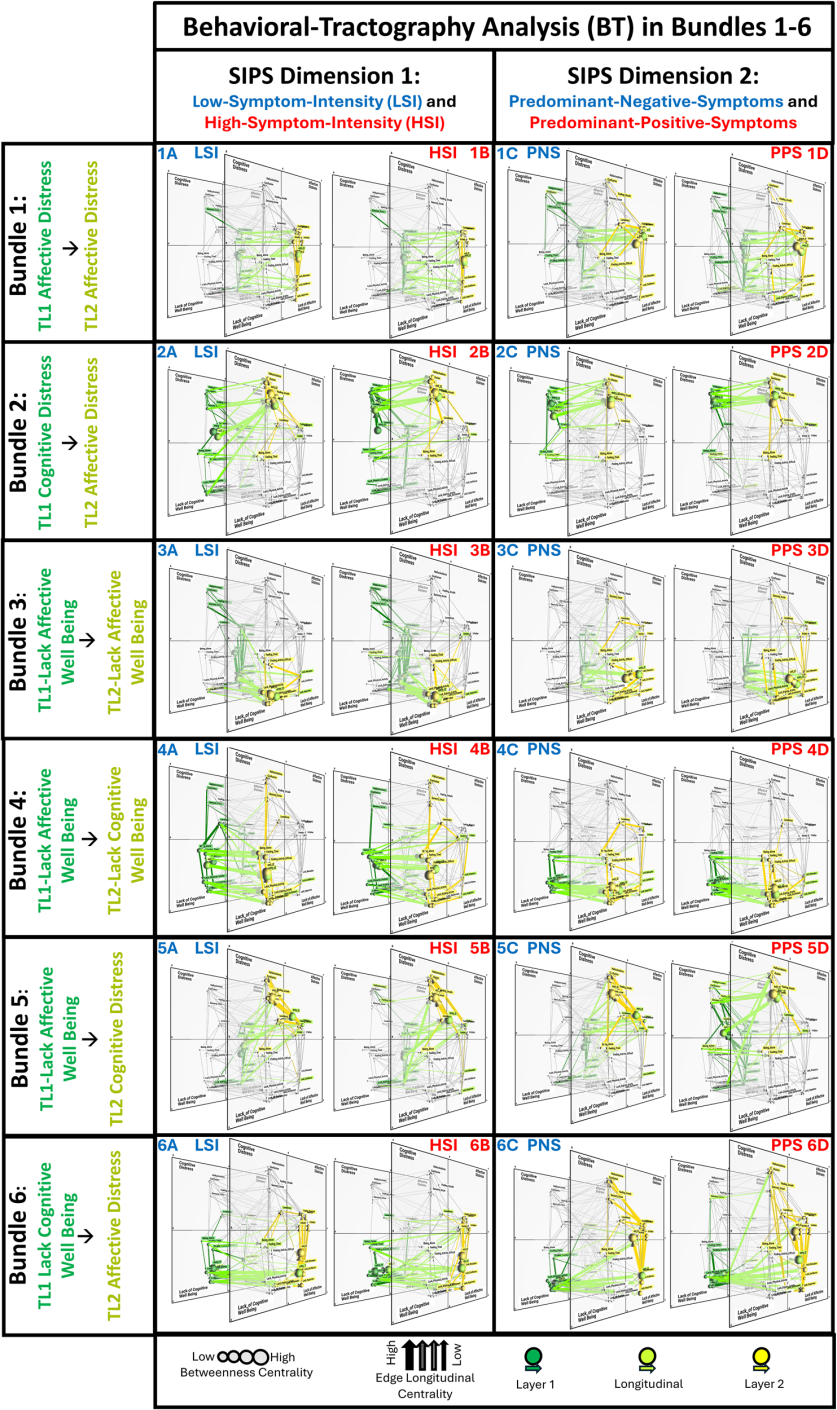
Behavioral Tractography Analysis across six bundles, describing consistent behavioral trajectories across High-vs-Low Intensity and Predominant Positive-vs-Negative subgroups. Figures have been derived using a dedicated open-source software (https://dev.mlnetwork-diplab.ch/) developed by the authors and available at (https://github.com/andreaimparato/Behavioral-Tractography-Toolbox/tree/main/MLNetwork). For each figure, we provide a link to an online platform allowing 3D interactive network visualization. Comparison of population-specific trajectories of Behavioral Tractography (BT) Bundles (1–6) in High-Symptom-Intensity vs Low-Symptom-Intensity subgroups defined according to SIPS-Dimension-1; and Predominantly-Positive-Symptoms vs Predominantly-Negative-Symptoms subgroups defined according to SIPS-Dimension-2. **1–6** Results of Behavioral-Tractography trajectory for each of 6 Bundles in High-Symptom-Intensity (**A**), Low-Symptom-Intensity (**B**), Predominantly-Positive-Symptoms (**C**) and Predominantly-Negative-Symptoms (**D**) subgroups; Green to Yellow color coding reflects the dynamic progressing of paths from TL1 to TL2 (Start-TL1 ➔ Exit-TL1: Dark Green; Exit-TL1 ➔ Entry-TL2: Light Green; Entry-TL2 ➔ End-TL2: Yellow). **1A–1D** Bundle 1: TL1-Affective-Distress➔TL2- Affective-Distress. **2A–2D** Bundle 2: TL1- Cognitive-Distress➔TL2-Affective-Distress. **3A–3D**: Bundle 3: TL1-Lack-Affective-Wellbeing➔TL2- Lack-Affective-Wellbeing. **4A–4D**: Bundle 4: TL1- Lack-Affective-Wellbeing➔TL2-Lack-Cognitive-Wellbeing. **5A–5D**: Bundle 5: TL1- Lack-Affective-Wellbeing➔TL2-Cognitive-Distress. **6A–6D**: Bundle 6: TL1- Lack-Cognitive-Wellbeing➔TL2-Affective-Distress. Links to 3D interactive visualization: HSI: https://dev.mlnetwork-diplab.ch/3dvisualizer/dim1_dim1_cl1/. https://dev.mlnetwork-diplab.ch/3dvisualizer/dim1_dim1_cl2/. https://dev.mlnetwork-diplab.ch/3dvisualizer/dim1_dim1_cl3/. https://dev.mlnetwork-diplab.ch/3dvisualizer/dim1_dim1_cl4/. https://dev.mlnetwork-diplab.ch/3dvisualizer/dim1_dim1_cl5/. https://dev.mlnetwork-diplab.ch/3dvisualizer/dim1_dim1_cl6/. LSI: https://dev.mlnetwork-diplab.ch/3dvisualizer/dim1_dim2_cl1/. https://dev.mlnetwork-diplab.ch/3dvisualizer/dim1_dim2_cl2/. https://dev.mlnetwork-diplab.ch/3dvisualizer/dim1_dim2_cl3/. https://dev.mlnetwork-diplab.ch/3dvisualizer/dim1_dim2_cl4/. https://dev.mlnetwork-diplab.ch/3dvisualizer/dim1_dim2_cl5/. https://dev.mlnetwork-diplab.ch/3dvisualizer/dim1_dim2_cl6/. PNS: https://dev.mlnetwork-diplab.ch/3dvisualizer/dim2_dim1_cl1/. https://dev.mlnetwork-diplab.ch/3dvisualizer/dim2_dim1_cl2/. https://dev.mlnetwork-diplab.ch/3dvisualizer/dim2_dim1_cl3/. https://dev.mlnetwork-diplab.ch/3dvisualizer/dim2_dim1_cl4/. https://dev.mlnetwork-diplab.ch/3dvisualizer/dim2_dim1_cl5/. https://dev.mlnetwork-diplab.ch/3dvisualizer/dim2_dim1_cl6/. PPS: https://dev.mlnetwork-diplab.ch/3dvisualizer/dim2_dim2_cl1/. https://dev.mlnetwork-diplab.ch/3dvisualizer/dim2_dim2_cl2/. https://dev.mlnetwork-diplab.ch/3dvisualizer/dim2_dim2_cl3/. https://dev.mlnetwork-diplab.ch/3dvisualizer/dim2_dim2_cl4/. https://dev.mlnetwork-diplab.ch/3dvisualizer/dim2_dim2_cl5/. https://dev.mlnetwork-diplab.ch/3dvisualizer/dim2_dim2_cl6/.

**Fig. 5:**
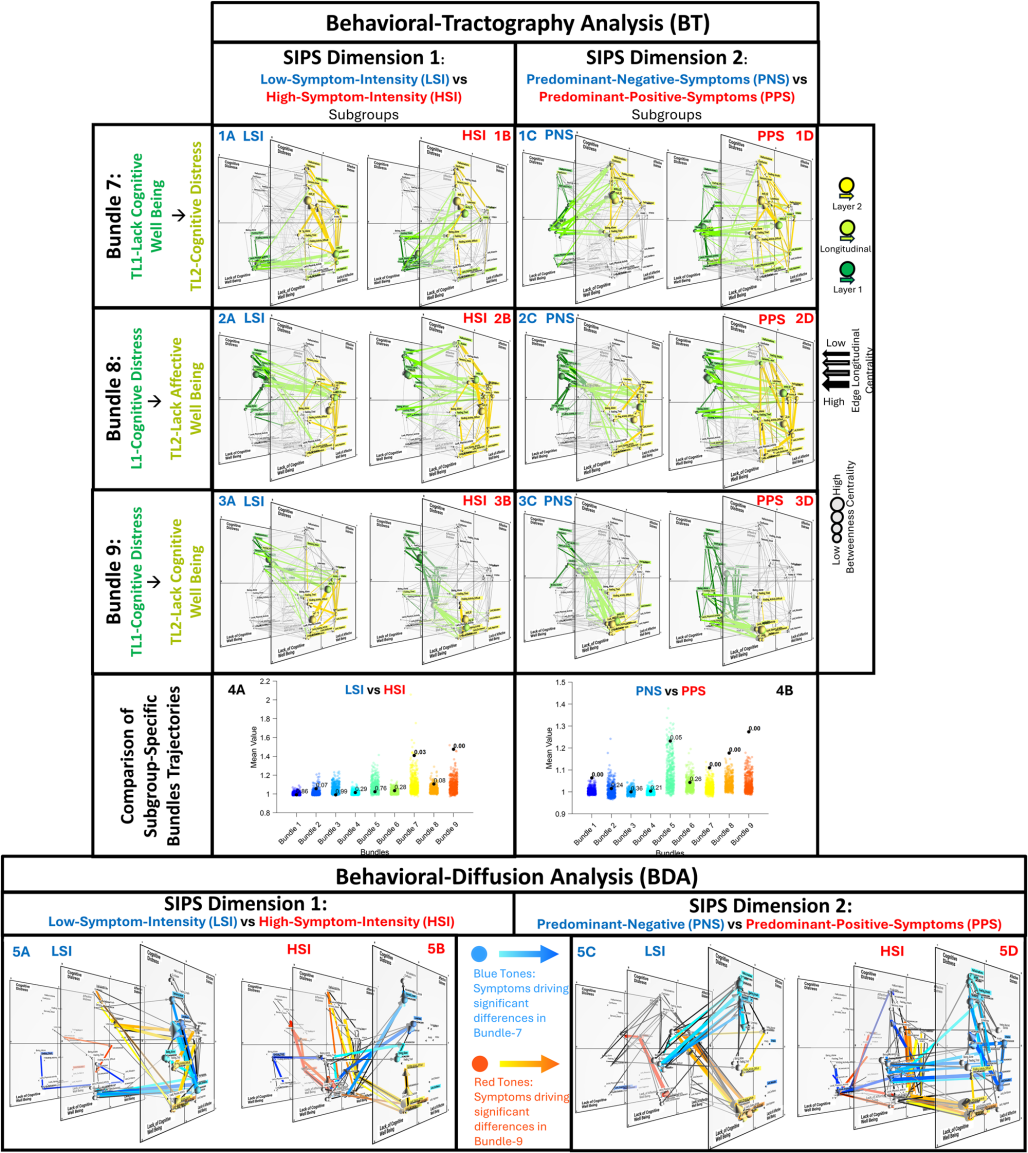
Behavioral Tractography and Diffusion Analysis across three bundles describing population-specific behavioral dynamics across High-vs-Low Intensity and Predominant Positive-vs-Negative subgroups. Figures have been derived using a dedicated open-source software (https://dev.mlnetwork-diplab.ch/) developed by the authors and available at (https://github.com/andreaimparato/Behavioral-Tractography-Toolbox/tree/main/MLNetwork). For each figure we provide a link to an online platform allowing 3D interactive network visualization. Comparison of population-specific trajectories of Behavioral-Tractography (BT) Bundles (7–9) in High-Symptom-Intensity vs Low-Symptom-Intensity subgroups defined according to SIPS-Dimension-1; and Predominantly-Positive-Symptoms vs Predominantly-Negative-Symptoms subgroups defined according to SIPS-Dimension-2. **1–3**: Results of Behavioral-Tractography trajectory for Bundles 7-9 in High-Symptom-Intensity (**A**), Low-Symptom-Intensity (**B**), Predominantly-Positive-Symptoms (**C**) and Predominantly-Negative-Symptoms(**D**) subgroups; Green–yellow coding indicates path progression from TL1 to TL2 (Start-TL1 ➔ Exit-TL1: Dark Green; Exit-TL1 ➔ Entry-TL2: Light Green; Entry-TL2 ➔ End-TL2: Yellow). **1A–1D**: Bundle 7: TL1- Lack-Cognitive-Wellbeing ➔TL2-Cognitive-Distress. **2A–2D**: Bundle 8: TL1-Cognitive-Distress➔TL2-Lack-Affective-Wellbeing. **3A–3D**: Bundle 9: TL1-Cognitive-Distress➔TL2- Lack-Cognitive-Wellbeing. **4A**, **4B:** Statistical comparison of average trajectories across nine BT-Bundles between High-Symptom-Intensity and Low-Symptom-Intensity (**A**)/Predominantly-Positive-Symptoms and Predominantly-Negative-Symptoms (**B**). For each bundle, we computed the ratio of between-group to within-group Euclidian distances between Bundle specific pathways across population. Significance was tested via 500-permutation null distribution; *p* values indicate the proportion of permutations exceeding observed ratios. Bundles with *p* < 0.05 differentiate populations while maintaining within-group consistency, reflecting distinct symptom-interaction dynamics. **5A–5D**: Results of Behavioral-Diffusion-Analysis (BDA) for variables in LSI (**5A**), HSI (**5B**), PNS (**5A**) and PPS (**5B**). Warm colors identify variables that drive significant BT differences in Bundle-9 (TL1- Lack-Cognitive-Wellbeing ➔TL2-Cognitive-Distress). Cold colors identify variables that drive significant BT differences in Bundle-7 (TL1-Cognitive-Distress➔TL2- Lack-Cognitive-Wellbeing). BDA trajectories reflecting differential functional role of TL1-Variables connect TL1 to TL2, while BDA trajectories reflecting differential functional role of TL2-Variables connected TL2 to TL3. The individual PC paths that contribute to average BDA trajectories are represented in Black. Links to 3D interactive visualization: HSI: https://dev.mlnetwork-diplab.ch/3dvisualizer/dim1_dim1_cl7/. https://dev.mlnetwork-diplab.ch/3dvisualizer/dim1_dim1_cl8/. https://dev.mlnetwork-diplab.ch/3dvisualizer/dim1_dim1_cl9/. https://dev.mlnetwork-diplab.ch/3dvisualizer/qpa_dim1_hsi/. LSI: https://dev.mlnetwork-diplab.ch/3dvisualizer/dim1_dim2_cl7/. https://dev.mlnetwork-diplab.ch/3dvisualizer/dim1_dim2_cl8/. https://dev.mlnetwork-diplab.ch/3dvisualizer/dim1_dim2_cl9/. https://dev.mlnetwork-diplab.ch/3dvisualizer/qpa_dim1_lsi/. PNS: https://dev.mlnetwork-diplab.ch/3dvisualizer/dim2_dim1_cl7/. https://dev.mlnetwork-diplab.ch/3dvisualizer/dim2_dim1_cl8/. https://dev.mlnetwork-diplab.ch/3dvisualizer/dim2_dim1_cl9/. https://dev.mlnetwork-diplab.ch/3dvisualizer/qpa_dim2_pns/. PPS: https://dev.mlnetwork-diplab.ch/3dvisualizer/dim2_dim2_cl7/. https://dev.mlnetwork-diplab.ch/3dvisualizer/dim2_dim2_cl8/. https://dev.mlnetwork-diplab.ch/3dvisualizer/dim2_dim2_cl9/. https://dev.mlnetwork-diplab.ch/3dvisualizer/qpa_dim2_pps/.

We then explored the potential of a recently proposed Behavioral-Tractography approach to dissect specific behavioral pathways linked to different SIPS symptom dimensions. Behavioral-Tractography performed on the entire 22q11DS sample, revealed nine distinct bundles composed of behavioral pathways mediating qualitatively similar dynamic behavioral interactions, as reflected by similar three-dimensional trajectories (see Supplementary Analysis 1 and Fig. 4 / Fig. 5 for subgroup-specific results). Both the composition and the average trajectory of such Behavioral-Tractography-Bundles were highly consistent with previous observations in independent typically developing and clinical samples (Supplementary Analysis 1).

The average trajectory of such Behavioral-Tractography-Bundles, described in detail in Supplementary Figure 1, provided a quantitative and graphical description of the main types of dynamic interactions between psychological states.

*Bundles 1-2* connected psychological distress states in the upper portion of the network across time.

*Bundle-1*: TL1-Affective-Distress → TL2-Affective-Distress, mediated by TL1-*Lacking Relaxation*, TL1-*Lacking Happiness*, TL2- *Lacking Relaxation*, TL2- *Sadness*.

*Bundle-2*: TL1-Cognitive-Distress → TL2-Affective-Distress, mediated by TL1- *Feeling Unsafe*, TL1- *Hallucinations*, TL2- *Hallucinations*

*Bundles 3-4* connected lack of psychological well-being states in the lower portion of the network across time.

*Bundle-3*: TL1-Lacking-Affective-Wellbeing → TL2-Lacking-Affective-Wellbeing, mediated by TL1-*Lacking-Happiness* and TL1-*Lacking Confidence*.

*Bundle-4*: TL1-Lacking-Affective-Wellbeing → TL2-Lacking-Cognitive-Wellbeing (LCWB), mediated by TL1-*Lacking Motivation*, TL2- *Lacking Physical Activity*, TL2- *Lacking Enjoying Activity*, TL2- *Lacking Concentration*.

*Bundles 5-7* connected lack of psychological well-being to psychological distress.

*Bundle-5*: TL1-Lacking-Affective-Wellbeing → TL2-Cognitive-Distress, mediated by TL1-*lacking Happiness*, TL1-*Feeling Rejected*, TL2-*Anxiety*, TL2-*Sadness*.

*Bundle-6*: TL1-Lacking-Cognitive-Wellbeing → TL2-Affective-Distress, mediated by TL2-*Lacking-Happiness*, TL2-*Lacking Confidence* and TL2-*Feeling Rejected*.

*Bundle 7*: TL1-Lacking-Cognitive-Wellbeing → TL2-Cognitive-Distress, mediated by TL1-Social Isolation (*Being Alone*, *Loneliness*), TL1/TL2-*Feeling Tired*, TL2-*Being Alone*

*Bundles 8-9* connected psychological distress to lack of psychological well-being.

*Bundle 8*: TL1-Cognitive-Distress → TL2-Lacking-Affective-Wellbeing, mediated by TL1-*Loneliness*, TL1- *Anxiety*, TL1-*Sadness*, TL1-*Being Alone*, TL2-*Lacking Relaxation*, TL2-*Anxiety*, TL2-*Sadness*.

*Bundle 9*: TL1-Cognitive-Distress → TL2-Lacking-Cognitive-Wellbeing mediated by TL1-*Lacking Relaxation*, TL1-*Irritation*, TL1-*Feeling Rejected*

We then performed a two-population Behavioral-Tractography, to identify population-specific behavioral trajectories differentiating SIPS-Dimension subgroups. We applied Behavioral-Diffusion-Analysis to pinpoint individual variables driving differences in Bundle trajectories across populations.

Results revealed that the trajectories of Bundles 1 through 6 were largely similar across SIPS-Dimension subgroups (see Fig. 4 and Fig. 5 Panel-4) and consistent with those described above for the entire sample. Both SIPS dimensions, however, differentially modulated the bi-directional behavioral pathways linking Lacking-Cognitive-Wellbeing (*Lacking-Enjoyment, Finding-Activity-Difficult, Lacking-Concentration, Lacking-Excitement, Lacking-Motivation)*and Psychotic/Cognitive Distress (*Lacking-Enjoyment, Finding-Activity-Difficult, Lacking-Concentration, Lacking-Excitement, Lacking-Motivation)*states, captured by population-specific trajectories for Bundles 7–9 (see Fig. 5).

Here we provide a brief description of such population-specific trajectories which is detailed in Fig. 5, along with links to a dedicated online 3D platform facilitating exploration of results. BDA results are detailed in Supplementary-Table 1 while Fig. 5 Panel-5 A/B/C/D depicts population-specific BDA vectors of variables, driving differences in Bundle Trajectories.

*Bundle-7*, connecting TL1-Lacking-Cognitive-Wellbeing, to TL2- Psychotic/Cognitive-Distress, was differentially modulated by both SIPS-Dimension-1 (*p* = 0.03) and SIPS-Dimension-2 (*p* < 0.001), defining 4 population specific trajectories.

*SIPS-Dimension-1-LSI* (Fig. 5 Panel-1A): Effects of TL1-Lacking-Cognitive-Wellbeing to TL-2-Psychotic/Cognitive-Distress indirectly mediated by TL2 Lacking-Affective-Wellbeing (TL2-*Lacking-Confidence*).

*SIPS-Dimension-1-HSI* (Fig. 5 Panel-1B): Direct pathway linking TL1-Lacking-Cognitive-Wellbeing (*TL1-Feeling-Tired*) to TL2 social isolation (TL2-*Loneliness*, TL2-*Being-Alone*) and TL2-Psychotic/Cognitive-Distress (TL2-*Sensory-Issue*).

*SIPS-Dimension-2-PNS* (Fig. 5 Panel-1C): TL1-Social-Isolation predicts TL2-Psychotic/Cognitive-Distress (TL2-*Feeling-Unsafe*, TL2-*Hallucinations*, TL2-*Confusing-Reality-with-Imagination)*.

*SIPS-Dimension-2-PPS* (Fig. 5 Panel-1D): TL1-Lacking-Cognitive-Wellbeing (TL1-*Lacking-Excitement*, TL1-*Lacking-Enjoyment*), mediates the effects of TL1-Social Isolation on subsequent TL-2 Affective variables (TL2-*Lacking-Relaxation*, TL2-*Irritation)* and TL2-Psychotic/Cognitive-Distress.

Taken together, HSI and PPS Bundle-7 trajectories, would suggest that in individuals with more severe SIPS positive symptoms, Lacking-Cognitive-Wellbeing variables exert a more direct effect on subsequent EMA Psychotic/Cognitive-Distress variables, compared to a higher contribution of previous social isolation in PNS subgroup, and subsequent affective variables in LSI. Interestingly, the differential contribution of Lacking-Cognitive-Wellbeing variables was observed despite the lower intensity with which these states were reported in both the HSI and PPS subgroups, potentially indicating heighted vulnerability to develop Psychotic/Cognitive-Distress following insufficient Cognitive-Wellbeing.

*Bundle-9*, which mediated the opposite behavioral pathway linking TL1-Psychotic/Cognitive-Distress to TL2-Lacking-Cognitive-Wellbeing was also was differentially modulated by both SIPS-Dimension-1 (*p* < 0.001) and SIPS-Dimension-2 (*p* < 0.001), defining 4 population-specific trajectories.

*SIPS-Dimension-1-LSI* (Fig. 5 Panel-3A): Effects of TL1-Psychotic/Cognitive-Distress on TL2-Lacking-Cognitive-Wellbeing, are mediated by TL1-Affective-Distress (*TL1-Anxiety*) and TL2-Lacking-Affective-Wellbeing.

*SIPS-Dimension-1-HSI* (Fig. 5 Panel-3B): Effects of TL1-Psychotic/Cognitive-Distress on TL2-Lacking-Cognitive-Wellbeing, are mediated by TL1-Lacking-Affective-Wellbeing (TL1-*Lacking-Relaxation*)

*SIPS-Dimension-2-PNS* (Fig. 5 Panel-3C): Effects of TL1-Psychotic/Cognitive- Distress on TL2-Lacking-Cognitive-Wellbeing. are mediated by TL1-Affective-Distress variables (*TL1-Sadness*).

*SIPS-Dimension-2-PPS* (Fig. 5 Panel-3D): Effects of TL1-Psychotic/Cognitive-Distress on TL2-Lacking-Cognitive-Wellbeing are mediated by TL1-Lacking-Affective-Wellbeing (TL1-*Lacking-Relaxation*).

Taken together, the similar Bundle-9 trajectories in HSI and PPS subgroups, would suggest that individuals with more severe SIPS positive symptoms experience more direct transitions from previous TL1-Psychotic/Cognitive-Distress to subsequent TL2-Lacking-Cognitive-Wellbeing, occurring independently of affective distress variables.

PNS individuals instead presented a unique pathway linking TL1-Sadness to TL2-Lacking-Cognitive-Wellbeing. A similar pattern contributed to the differential PNS trajectory of BT-Bundle-8 (Fig. 5 Panel-2C), where TL1-*Sadness* (*p* < 0.001) mediated the transition from TL1-Psychotic/Cognitive-Distress and TL2-Lacking-Affective-Wellbeing. This would suggest that individuals with PNS, affective-Distress variables, in particular *Sadness*, play a more direct role in the severe reductions of psychological well-being reported by this population. Interestingly, this was observed despite the reduced intensity of *Sadness* reported in the PNS subgroup, suggesting that PNS individuals may present heightened vulnerability to severe psychological wellbeing reductions in response to relatively minor affective distress experiences. Overall, these results would suggest that the average intensity of psychological experiences may provide an incomplete, or even misleading representation of their importance in influencing fluctuations in future psychological states.

## Discussion

In the present paper, we explore the potential of a recently proposed Behavioral-Tractography approach for translating the rich dynamic and ecologically grounded view of psychological phenomena provided by EMA^15^, to a deeper and clinically meaningful understanding of the pathophysiological interplay between different psychosis symptom dimensions^12,14^.

We firstly evaluated weather EMA questionnaires could effectively capture the same multidimensional clinical phenomena characterized with gold-standard psychosis clinical interviews in 22q11DS. Indeed, such validation is a critical perquisite for drawing conclusions on psychosis symptom dynamics from EMA studies^14^. The structure of across subject variation in SIPS symptom intensity was highly consistent with previous reports in 22q11DS^42,43^. SIPS-Dimension-1 reflected overall intensity of both positive and negative symptoms and the propensity for these symptoms to manifest as combined clinical presentations in the more severely affected High-Symptom-Intensity (HSI) subgroup. When accounting for overall disease severity, SIPS-Dimension-2 highlighted the differential propensity for individuals to manifest with either predominantly-positive vs. predominantly-negative symptom patterns.

The average intensity of most EMA items significantly differed across such SIPS-Dimensions. Individuals with High-Symptom-Intensity (HSI) and Predominantly-Positive-Symptom (PPS) both reported higher EMA items reflecting positive psychotic symptoms (e.g., *Hallucinations*, *Feeling-Unsafe, Confusing-Reality-with-Imagination*), while individuals with Predominantly-Negative-Symptoms (PNS) reported reduced psychological well-being—both affective (e.g., *Lacking-Happiness Lacking-Relaxation*) and cognitive/motivational (e.g., *Lacking-Motivation, Lacking-Enjoyment*). These results show that EMA questionnaires can effectively capture across-subject variation in both positive and negative psychosis symptomatic dimensions, defined with gold standard clinical interviews.

To further evaluate EMA questionnaire validity, we explored whether traditional psychosis dimensions would map onto the low-dimensional structure of dynamic psychological phenomena, characterized by EMA Network-Dimensionality-Reduction (NDR)^32^. NDR results were highly consistent with previous literature^32^. The correlation structure of behavioral fluctuations was organized along two rationality and valence dimensions, defining four Affective-Distress, Cognitive-Distress, Lacking-Affective-Wellbeing and Lacking-Cognitive-Wellbeing network quadrants. Remarkably, the ways in which EMA items differed according to SIPS dimensions, was mirrored by their differential mapping along such NDR dimensions. EMA-correlates of positive symptoms clustered in the upper-left Cognitive-Distress quadrant, while correlates of negative symptom clustered in distinct lower-left Lacking-Cognitive-Wellbeing and lower-right Lacking-Affective-Wellbeing quadrants. The alignment between SIPS-EMA correlations and NDR dimensions, which can be defined as trait-state homomorphy^48^, suggests that latent factors underlying inter-individual variability of psychotic symptoms severity may be tied to mechanisms underpinning dynamic fluctuations in symptom severity experienced by individual patients across time. This novel observation could carry significant implications for trait-vs-state models of psychosis pathophysiology^49,50^. Specifically, it would suggest that psychosis symptoms intensity, characterized by traditional clinical assessments, does not measure a static level of disease severity, but rather reflects the aggregate of inherently dynamic fluctuations in different symptom dimensions^49,50^. Our results suggest that EMA questionnaires can effectively capture dynamic fluctuations in the same phenomena measured with current gold-standard static psychosis assessments^14^.

The second objective of the present study was to explore EMA’s potential in generating additional clinically relevant insights on behavioral dynamics associated with different psychosis symptom dimensions. We employed an innovative 3D-Temporal-Multilayer-Network approach, providing an integrated representation of cross-sectional co-occurrence and dynamic symptom interactions unfolding across time, which revealed distinct behavioral dynamics associated with SIPS positive symptom severity. Specifically, both HSI and PPS subgroups presented stronger correlations between EMA Psychotic/Cognitive-Distress items, while associations between psychotic and affective distress variables were weaker. This suggests that in more severely affected individuals, psychotic experiences (e.g., hallucinations, paranoia, reality confusion), are not only more intense, but also manifest as more multifaceted symptom constellations, that are increasingly dissociated from other forms of psychological distress. The increasing dissociation of cognitive and affective distress variables with increasing psychosis severity supports the value of the cognitive-to-affective *rationality* NDR dimensions in differentiating psychosis from other forms of psychological distress. Indeed, mapping of psychotic symptoms as cognitive distress, strikingly recapitulates phenomenological descriptions of the psychotic symptoms as a “cognitive” attempt to make sense of internally or externally generated events that are experienced as unusual, bizarre, or unexpected^51^.

The 3D-Temporal-Multilayer-Network approach further reveled that positive symptom severity exerted a combined impact on both cross-sectional correlations, and dynamic symptom interactions occurring from one assessment to the next. Indeed, HSI and PPS subgroups presented strengthening of longitudinal connections linking psychotic symptoms across time, suggesting that in more symptomatic subjects, psychotic symptoms become increasingly self-sustaining and independent from affective or contextual fluctuations. Interestingly, these results are highly consistent with Bayesian models of psychosis, according to which reality distortion underlying positive symptoms may stem from as an inability to flexibly adapt internal models of the world, based on dynamically fluctuating experience of recent contextual factors^52^. Of note, according to Bayesian models, individuals with ASD may present an opposite tendency to rely more strongly on recent sensory information^53–55^, which would prove advantageous in repetitive environments requiring high sensory precision^56^, but may also contribute to higher psychological volatility in response minor variation in contextual factors^55^. In accordance with such models, we recently demonstrated that ASD and 22q11DS populations present opposite network alterations, compared to typically developing individuals, reflecting diametrical differences in psychological-contextual dynamics. Our current results further support such Bayesian models, by demonstrating that differences in psychological-contextual dynamics observed across 22q11DS and ASD populations accentuate as psychosis severity increases within the 22q11DS population. Overall, these results suggest that measuring the short-term dynamics of psychosis phenomenology can provide additional insights on both the subjective experience and underlying pathophysiology of positive psychotic symptoms.

The unique comprehensive clinical assessment and analysis pipeline revealed dynamic behavioral alterations, which were not limited to positive symptoms, and specifically reflected SIPS negative symptom severity. Indeed, both HSI and PNS subgroups, presented stronger connections between EMA-correlates of negative symptoms (Lacking-Affective-Wellbeing and Lacking-Cognitive-Wellbeing variables in lower-left and lower-right quadrants), and Affective-Distress states (*Sadness, Irritation, Anxiety*, upper-right quadrant). This would suggest that in individuals with more severe negative symptoms, reductions of psychological well-being are not only more severe, but also more tightly related to concomitant affective distress. HSI and PNS subgroups also presented a widespread strengthening of time-lagged connections, which primarily affected “diagonal” connections linking different symptomatic dimensions across temporal layers. This would suggest that negative symptom severity may be linked to increased temporal dependencies between past and future psychological states, which, however, did not reflect the temporal inertia of more inherently stable states, but rather stemmed from an increased propensity to dynamically shift from one symptomatic dimension to another across time.

We employed the Behavioral Tractography pipeline to shed on such population-specific pathways underlying dynamic shifts between different symptom dimensions^32^. Interestingly, both SIPS Dimensions influenced bi-directional interactions between Cognitive-Distress positive symptoms and Lacking-Cognitive-Wellbeing negative symptoms, as captured by population-specific trajectories of Behavioral-Bundles 7 and 9. The HSI subgroup, characterized by a combined increase of positive and negative symptoms, presented more direct bidirectional trajectories between such states, while in the LSI subgroup, such interactions were mediated by affective variables. This would suggest the combined presentation of positive and negative symptoms may stem from a tighter dynamic interaction between states, rather than from the co-occurrence of independent disease mechanisms. Of note, this tighter relationship was not reflected in the strength of cross-sectional correlations, suggesting that individuals with combined positive and negative symptomatology still experience these symptoms as phenomenologically distinct events, occurring at different moments in time. Instead, the HSI subgroup presented heighted propensity to develop *secondary* psychotic experiences following previous motivational deficits, as well as secondary motivational deficits following previous psychotic cognitive distress.

The fine-grained Behavioral-Tractography pipeline further revealed a differential modulation of dynamic pathways linking EMA positive and negative symptoms, exerted by SIPS Predominantly-Positive vs Predominantly-Negative symptom patterns. In the PNS subgroup, the interaction between previous Lacking-Cognitive-Wellbeing and future Cognitive-Distress, described by Bundle-7, was more strongly mediated by social isolation factors, compared to a more direct effect of Lacking-Cognitive-Wellbeing, observed in PPS individuals. As such, in individuals with more severe positive symptoms, fluctuations in psychotic symptom severity may be more independent of contextual factors, in accordance with previously highlighted Bayesian models^52^. The PNS subgroup was instead characterized by direct pathways linking previous feelings of sadness associated with psychotic/cognitive-distress to subsequent reductions in affective and cognitive well-being states, described by Behavioral-Bundles 8 and 9. These results would suggest that increased negative symptom severity may stem from a heightened propensity to develop secondary reductions in motivation and affective well-being, following previous affective distress. Of note, these findings would challenge the view of negative symptoms as stable expressions of disease progression, that become increasingly intractable as their severity increases^5,57,58^. Indeed, not only did negative symptom intensity fluctuate at short-time frames, but such symptomatic dynamics were accentuated in more severely affected individuals, due to stronger modulation exerted by previous affective distress experiences.

To our knowledge, this is the first study to comprehensively investigate dynamic short-term interactions between the different symptom dimensions of psychosis. Our results are however consistent with a growing body of literature demonstrating that both positive and negative symptom dimensions can fluctuate dynamically in relation to external contextual factors and other psychological states^20,22,59–62^. Specifically, our results align with recent EMA studies^20,22,60,61^, suggesting that positive and negative symptoms can interact at much faster temporal dynamics than was traditionally conceptualized^47^, which could carry important pathophysiological implications. Indeed, current neurobiological models struggle to reconcile the proposedly opposite hyper-dopaminergic underpinnings of positive symptoms, with the dopaminergic hypoactivity thought to contribute to the motivational deficit dimension of negative symptoms^10^. Our results suggest that individuals presenting with combined positive symptoms and motivational deficits do not actually experience these symptoms concomitantly, but rather sequentially, through short-term temporal interactions. This could suggest that their opposite hyper/hypo dopaminergic underpinnings might potentially emerge from homeostatic regulation of striatal dopaminergic signaling^63–65^ that has been shown to play a key role in rapidly fluctuating secondary motivational deficits^66^.

At a broader level, our findings suggest that vulnerability to downstream psychological consequences may vary independently of the average intensity with which symptoms are experienced. Indeed, HSI and PPS individuals presented increased vulnerability to develop secondary positive psychotic symptoms following previous motivational deficits, despite reporting, on average, milder motivational symptoms. Similarly, in PNS individuals, vulnerability to develop secondary negative symptoms following previous affective distress was observed despite reduced intensity with which sadness was reported, suggesting heightened predisposition to develop psychological consequences of affective distress, rather than an increased exposure to such experiences. This would suggest that measuring dynamic behavioral interactions could be critical to dissect mechanisms underpinning not only severity but also differential vulnerability to psychological experiences across subjects^67^. From a clinical perspective, assessing behavioral dynamics could be essential to capture the relative importance of psychological variables in influencing fluctuations in future psychological states^68^.

From a clinical perspective, these findings would hence support the added value of EMA approaches for improving assessment and management of psychosis^16^. However, increasing the precision and detail of psychiatric assessments carries a concrete risk of drowning clinicians in information that is ultimately too complex to reliably inform clinical reasoning^69,70^. It has been suggested that in current practice, clinicians confront such complexity by flexibly combining a large-scale analysis of broad clinical patterns, similar to identifying the outline of the forest, with a second higher-level analysis of the role of individual clinical or contextual factors^71^. The Behavioral-Tractography pipeline is specifically designed to accommodate this process, which could potentially contribute to increase interpretability and clinical value of EMA. NDR dimensions provide a novel large-scale mapping of dynamic psychological fluctuations, that was both sufficiently conserved to allow intuitive comparisons across clinical populations, and sufficiently detailed to differentiate psychosis symptom dimensions within the 22q11DS sample. Behavioral-Tractography builds upon this to derive a unique multi-scale view of both the main types of dynamic behavioral interactions unfolding in daily life and of the role individual symptoms. In a previous study, we demonstrated that Behavioral-Tractography can differentiate 22q11DS individuals, with a known ground-truth genetic differences, from controls and individuals with idiopathic ASD^32^. Here, we demonstrate the potential of Behavioral-Tractography to provide additional insight on psychological contextual dynamics underpinning different symptom dimensions within the 22q11DS population. These results support the value of Behavioral-Tractography for elucidating differential pathophysiology of conditions that might otherwise appear homogenous based on current retrospective psychiatric assessments, which could potentially contribute to improved clinical management^67,72^.

## Methods

### Sample

68 22q11DS carriers (31% female, mean age = 19.34, SD = 5.18) were included in this study and recruited through the 22q11DS Swiss longitudinal cohort^41,73^. The majority (84% of subjects) overlapped with the 22q11DS sample described in the previously cited Behavioral-Tractography study, while 19% of participants of the previous study who lacked full clinical assessment were excluded here^32^. All participants had a confirmed genetic diagnosis of microdeletion 22q11.2 (determined by fluorescence in situ hybridization, multiplex ligation-dependent probe amplification, or micro-array analysis). Demographic details are described in Table 1.

The presence of DSM-5 psychiatric disorders was assessed with the Diagnostic Interview for Children and Adolescents-Revised^74^ or Schedule for Affective Disorders and Schizophrenia for School-Age Children Present and Lifetime Version^75^ (K-SADS-PL DSM-5) for participants under 18 years old and Structured Clinical Interview for DSM-IV Axis I (SCID-I)^76^ or DSM-V (SCID-5-CV)^77^ for participants above 18 years old.

The presence and severity of positive, negative, disorganization, and general symptoms of psychosis were evaluated along 7-point Likert scale (0–6), using the Structured Interview for Psychosis-Risk Syndromes^78^. We considered only items that were reported with non-zero intensity in >10% of patients, which resulted in excluding the Grandiosity (P3) item of the SIPS. This aligns with established literature^14^, which suggests that grandiosity is rarely reported by patients with 22q11 deletion syndrome.

### Ecological momentary assessment

EMA was performed with the RealLifeExp smartphone application^79^. The protocol, which was described in previous publications [13], consisted of semi-random notifications delivered eight times daily between 7:30 AM and 10:00 PM over a six-day period (maximum of 48 prompts). A minimum interval of 30 min was scheduled between consecutive notifications. Participants were granted a maximum of 15 min to initiate the questionnaire and unlimited time for completion. In the current study, in total 20 items, evaluated on a 7-point Likert scale were used.

The EMA questionnaire provided a comprehensive assessment of participants’ psychological states. It assessed psychological distress (*Sadness, Anxiety, Anger*) and wellbeing (*Happiness, Confidence, Excitement, Relaxation, Motivation*), with wellbeing items reverse-coded for analysis. It also assessed the presence of psychotic-like experiences (*Hallucinations, Confusing-Reality-with-Imagination, Feeling-Unsafe*). Additionally, participants reported contextual details such as social setting (*Being-Alone* vs with others) and activity (current task). They also rated their experiences of social context (*Feeling-Lonely, Feeling-Rejected)* and activity (*Enjoyment, Concentration, Struggling*). See Supplementary Material for the full EMA questionnaire.

### Data analysis

#### Correlation between SIPS clinical patterns and EMA item intensity

We started by characterizing the correlation between clinical patterns measured with the gold-standard SIPS clinical interview and the intensity of psychological states measured in everyday life with EMA.

Previous EMA validation studies have correlated specific EMA items with the severity of the corresponding symptoms measured with traditional clinical interviews^79^. For instance, the intensity of EMA-estimated hallucinations has shown to correlate with SIPS gold-standard clinical assessment of hallucination severity^79^.

Here, we aimed to provide a more comprehensive data-driven view of the correspondence between multidimensional clinical patterns measured with SIPS and psychological phenomena measured with EMA. To this end, we firstly applied Principal Component Analysis (PCA) implemented with the MATLAB (MATLAB Version: R2024a) *pca* function, to the SIPS data. We retained PCA dimensions that explained a significant portion (>10%) of the total variance, capturing coherent clinical patterns across subjects. We derived SIPS-Dimension scores capturing the extent to which the corresponding SIPS clinical pattern was expressed in each participant, which we correlated with the average intensity of each EMA item. We also applied k-means clustering using the MATLAB *k-means* function to identify subgroups that differentially expressed such SIPS-Dimensions and compared both SIPS and EMA item intensity across the resulting subgroups using a two-sample t-test.

#### Characterization of EMA behavioral dynamics through Behavioral Tractography

We then investigated whether differences in SIPS clinical patterns would also be associated with differences in patterns of dynamic interaction between behavioral states measured with EMA.

To this end, we employed a recently developed Behavioral Tractography framework which provides a quantitative and intuitive characterization of behavioral dynamics^32^. The BT framework is described in detail in a separate publication and is an open-source toolbox https://github.com/andreaimparato/Behavioral-Tractography-Toolbox, while a brief overview of the main steps of the analysis pipeline is provided below.

We first estimated the strength of associations between pairs of EMA items as the slope coefficient of a Linear Mixed-Effects Regression (MMLR) including a random intercept to account for across-subject variation in item intensity.

We estimated association strength between cross-sectional variables from the same EMA assessment as the slope coefficient of a Linear Mixed Regression (LMR) with a random intercept for each subject; formalized as follows: ${Y}_{{ij}}=\,{\beta }_{0}+\,{{\boldsymbol{\beta }}}_{{\boldsymbol{1}}}{X}_{{ij}}+{u}_{j}+{\varepsilon }_{{ij}}$ where ${Y}_{{ij}}$ is the outcome variable for subject *j* at observation *i*, ${X}_{{ij}}$ is the predictor variable for subject *j* at observation *i*, ${\beta }_{0}$ is the fixed intercept, ${\beta }_{1}$ is the fixed slope, ${u}_{j}$ is the random intercept, ${\varepsilon }_{{ij}}$ is the residual error term, using the *lme* function in MATLAB. By computing MMLR for each pair of EMA items we constructed a 20×20 adjacency matrix. Associations that were not significant at *p* < 0.05 after Benjamini–Hochberg multiple comparisons correction^80^ were set to 0. This matrix can be thought of as a network in which nodes are EMA items, and the edges capture the association strengths between nodes.

Next, we applied a Network Dimensionality Reduction (NDR) technique^29^ based on PCA, implemented through MATLAB *pca* function, to the above-mentioned adjacency matrix, in order to identify large-scale dimensions capturing the differential propensity for EMA items to manifest together. The respective network nodes for each of the EMA items were then positioned in a two-dimensional space according to their loading along the two main NDR dimensions. We estimated the accuracy of such 2-dimensional mapping as the negative correlation between the Euclidean distance separating network nodes and the association strength between corresponding EMA items.

We then built upon such 2-dimensional mapping of cross-sectional associations to develop a 3-dimensional view of the dynamics of interactions between EMA items unfolding across time. Analogous to the cross-sectional associations, we measured the strength of associations between EMA items across consecutive assessments using Time-lagged MMLR models. We then integrated these lagged associations with the previously identified cross-sectional relationships, yielding a comprehensive 40 × 40 multi-layer adjacency matrix that captured both current and lagged associations between variables, which could then be graphically represented as a 3-dimensional multi-layer network (3D-MLN). Cross-sectional connections were represented in separate temporal layers in which nodes/symptoms were positioned along *X*/*Y* axes according to their differential propensity to manifest together, as defined by NDR. Temporal Layers were separated along a temporal Z axis, which was populated by longitudinal time-lagged edges connecting EMA items from one temporal assessment to the next. These 3D-MLN were represented with a dedicated open-source network (mlnetwork-diplab.ch) visualization software that is integrated with the BT analysis toolbox. For the main figures of the present study, we included a link to the software allowing interactive 3D manipulation of networks, hence facilitating in depth exploration of the findings.

Aside from visual intuitiveness, the 3D representation also provided analytical advantages for quantitative characterization of behavioral dynamics. Specifically, by applying Graph-Theory algorithms to these 3D-ML networks, we characterized shortest paths connecting EMA variables across consecutive temporal layers. The 3D trajectory of such shortest paths could be decomposed in 4 sets of XYZ coordinates of its TL1-starting-node, TL1-exit-node, TL2-entry-node, and TL2-ending-node. We then applied k-means clustering to these coordinates in order to dissect “bundles” of behavioral pathways that shared similar 3D trajectories, reflecting similar roles in mediating dynamic transitions between behavioral states. This approach was conceptually inspired by the Neuroimaging Tractography analysis dissecting white-matter bundles pathways based on 3D water diffusion direction in axonal tracts^81^. Moreover, Behavioral Diffusion Analysis (BDA) provided a micro-scale characterization of the contribution of individual variables to distinct behavioral bundles, which we estimated quantitatively as the average 3D diffusion direction of paths that traversed each network node. As such, Behavioral-Tractography allows for a flexible navigation of behavioral dynamics from both a large-scale and detailed symptom-level perspective.

#### Correlation between SIPS clinical patterns and dynamic interaction between EMA items in daily life

Next, we explored whether Behavioral-Tractography could identify differences in EMA psychological dynamics corresponding to the differential clinical patterns described by the SIPS.

We firstly investigated how SIPS-PCA dimensions moderated the strength of associations between individual pairs of EMA variables, which was achieved by adding each subject’s SIPS-PCA score to the MMLR and focusing on the interaction term between this subject-specific SIPS-PCA score and the strength of association between EMA variables. The model was formalized as follows: ${Y}_{{ij}}=\,{\beta }_{0}+\,{\beta }_{1}{X}_{{ij}}+\,{\beta }_{2}{S}_{j}+\,{{\boldsymbol{\beta }}}_{{\boldsymbol{3}}}\left({X}_{{ij}}* {S}_{j}\right)+{u}_{j}+\,{\varepsilon }_{{ij}}$ where ${Y}_{{ij}}$ is the outcome variable for subject *j* at observation *i*, ${X}_{{ij}}$ is the predictor variable for subject *j* at observation *i*, ${S}_{j}$ is subject-specific score (SIPS-PCA dimension score), same for all observations of subject j, ${X}_{{ij}}* {S}_{j}$ is interaction term between the EMA predictor and the subject-specific score, ${\beta }_{0}$ is the fixed intercept, ${\beta }_{1}$ is the fixed slope, ${\beta }_{2}$ fixed effect for subject-specific score, ${{\boldsymbol{\beta }}}_{{\boldsymbol{3}}}$ effect of the interaction between the EMA predictor and the subject-specific score, ${u}_{j}$ is the random intercept per subject, ${\varepsilon }_{{ij}}$ is the residual error term. We repeated this analysis for both SIPS-PCA dimensions and employed the 3D-MLN view to graphically represent both contemporaneous and time-lagged connections that were significantly moderated by SIPS-PCA scores after Benjamini–Hochberg multiple comparisons correction^80^ See Fig. 3.

We then employed Behavioral-Tractography to provide a meta-scale representation of how SIPS-PCA dimensions influenced dynamic behavioral interactions between EMA variables. First, subjects were grouped into clusters in SIPS-PCA space with k-means clustering as described previously. Separate networks were constructed in each sub-population for which we computed population-specific shortest-paths connecting past to future behavioral variables across temporal layers. We then applied a modified k-means clustering procedure to pathway coordinates measured in both sub-populations (Start-TL1, Exit-TL1-Population-A, Exit-TL1-Population-B, Entry-TL2-Population-A, Entry-TL2-Population-B, End-TL2). The resulting bundles connected a shared set of TL1 to TL2 variables across populations, while still allowing for potential population-specific differences in behavioral trajectories. Such differences in 3D trajectories would suggest qualitative differences in dynamic behavioral interactions between subsets of EMA variables. To assess significance, we then compared these differences in BT-bundle trajectories against a null distribution obtained by permuting subjects randomly across populations.

## Supplementary information

SupplementaryMaterial

## Data Availability

The full data are freely available at https://github.com/andreaimparato/Behavioral-Tractography-Toolbox.

## Code availability

The code used in the analysis is freely available at https://github.com/andreaimparato/Behavioral-Tractography-Toolbox.
