# Supplementary material for: Shedding light on the dynamic interplay of positive and negative symptoms of psychosis with Behavioral Tractography
Source: NPJ Digit Med. 2025 Nov 27;8:759. doi: 10.1038/s41746-025-02128-6 (PMC12705817; doi:10.1038/s41746-025-02128-6)
Supplement: Supplementary file 1 — SupplementaryMaterial [file 41746_2025_2128_MOESM1_ESM.pdf]

SUPPLEMENTARY MATERIAL INDEX

- **Supplementary Table 1. Behavioral Diffusion Analysis (BDA)**
- **Supplementary Analysis 1: Replication of Behavioral Tractography and Diffusion Analysis in 22q11DS and Typically Developing Sample**
- **EMA Protocol**
- **References**

Supplementary Table 1. Behavioral Diffusion Analysis (BDA). Detailed quantitative comparison of BDA-derived trajectories across samples, highlighting how specific symptoms contribute to behavioral dynamics. The columns (CL1–CL9) represent distinct bundles derived from the Behavioral-Tractography analysis. The table is organized into the two SIPS-PCA derived dimensions: High-/Low-Symptom-Intensity Dimension and Predominant Positive/Negative Symptom Dimension.
The first row (BT-P-values) of the table presents the global p-values for the differences in BT-bundle trajectories between samples, tested against a null distribution obtained by randomly permuting subjects across populations. The subsequent rows display the BDA analysis p-values, which are organized by Temporal Layers 1 and 2. These reflect the statistical significance of specific symptom contributions to behavioral dynamics within each bundle and temporal layer.

High-/ Low-Symptom-Intensity Dimension

	BT-P-values	CL1 0.86	CL2 0.07	CL3 0.99	CL4 0.29	CL5 0.76	CL6 0.28	CL7 0.03	CL8 0.08	CL9 0.00
	**EMA Symptoms**	**CL1**	**CL2**	**CL3**	**CL4**	**CL5**	**CL6**	**CL7**	**CL8**	**CL9**
TEMPORAL LAYER 1	Lack Relaxation	0.84	1.00	0.81	0.99	0.75	0.42	0.63	0.56	0.00
	Loneliness	1.00	0.70	0.73	0.15	1.00	0.88	1.00	0.39	0.10
	Anxiety	0.90	0.45	0.89	0.65	0.81	1.00	1.00	0.99	0.03
	Lack Happiness	0.47	0.01	0.82	0.25	0.24	0.04	0.31	1.00	0.33
	Irritation	0.30	1.00	0.12	0.13	0.30	1.00	0.28	0.76	0.25
	Sensory Issue	0.97	0.23	0.96	0.32	1.00	1.00	1.00	0.97	0.21
	Lack Excitement	0.18	1.00	0.07	1.00	0.62	0.59	0.45	1.00	0.06
	Sadness	0.88	0.00	0.88	0.32	0.45	0.15	1.00	0.98	0.07
	Lack Confidence	0.97	1.00	0.08	0.12	0.93	1.00	1.00	1.00	1.00
	Feeling Rejected	0.31	0.90	0.75	1.00	0.93	1.00	1.00	0.07	0.28
	Feeling Unsafe	1.00	0.58	0.52	1.00	0.88	1.00	1.00	0.22	0.46
	Confusion	1.00	0.18	0.95	0.71	1.00	1.00	1.00	0.06	0.95
	Hallucinations	1.00	0.00	0.72	0.84	0.65	1.00	1.00	0.33	0.22
	Feeling Tired	1.00	0.85	0.50	0.15	1.00	0.33	0.05	0.44	0.40
	Lack Motivation	1.00	0.09	0.65	0.14	1.00	0.41	0.65	1.00	1.00
	Lack Physical Activity	1.00	0.68	1.00	0.03	1.00	0.03	0.33	1.00	1.00
	Finding Activity Difficult	1.00	0.94	1.00	0.75	1.00	0.91	0.49	0.38	1.00
	Lack Enjoying Activity	1.00	1.00	1.00	0.77	0.17	0.84	0.58	1.00	1.00
	Lack Concentration	1.00	0.77	1.00	0.52	1.00	0.06	0.12	1.00	1.00
	Being Alone	1.00	0.07	1.00	0.01	1.00	0.24	1.00	0.10	1.00
	**Symptoms**	**CL1**	**CL2**	**CL3**	**CL4**	**CL5**	**CL6**	**CL7**	**CL8**	**CL9**
TEMPORAL LAYER 2	Lack Relaxation	0.54	1.00	0.76	0.21	1.00	0.50	0.60	0.44	0.06
	Loneliness	1.00	0.56	1.00	0.90	0.00	0.36	0.00	0.23	1.00
	Anxiety	0.56	0.64	0.95	0.47	0.52	0.04	0.18	0.33	0.40
	Lack Happiness	0.89	1.00	0.97	0.34	0.99	0.81	0.98	0.40	0.91
	Irritation	0.13	0.15	1.00	0.09	1.00	0.11	0.09	0.14	0.13
	Sensory Issue	1.00	0.14	1.00	0.63	0.00	1.00	0.00	0.98	0.98
	Lack Excitement	1.00	1.00	0.11	0.21	1.00	0.05	1.00	0.00	0.04
	Sadness	0.92	0.16	1.00	1.00	0.28	0.16	0.07	0.00	0.93
	Lack Confidence	0.88	1.00	1.00	0.99	1.00	0.00	0.00	0.09	0.10
	Feeling Rejected	0.96	0.94	1.00	1.00	0.96	0.09	0.07	0.92	1.00
	Feeling Unsafe	1.00	0.00	1.00	1.00	0.02	1.00	0.15	1.00	1.00
	Confusion	1.00	0.89	1.00	0.71	0.10	1.00	0.10	0.99	1.00
	Hallucinations	1.00	0.27	1.00	0.84	0.00	1.00	0.09	0.16	1.00
	Feeling Tired	1.00	0.75	0.51	0.91	1.00	1.00	0.55	0.93	0.11
	Lack Motivation	1.00	1.00	0.22	0.27	1.00	1.00	0.99	0.12	0.04
	Lack Physical Activity	1.00	1.00	0.13	0.20	1.00	1.00	1.00	1.00	1.00
	Finding Activity Difficult	1.00	1.00	0.01	0.99	1.00	1.00	0.87	0.52	0.00
	Lack Enjoying Activity	1.00	1.00	0.03	0.26	1.00	0.99	0.96	0.05	0.00
	Lack Concentration	1.00	1.00	0.11	0.06	1.00	1.00	0.99	0.03	0.03
	Being Alone	1.00	0.69	1.00	0.54	1.00	0.66	0.03	1.00	1.00

Predominant Positive- negative Symptom Dimension

	BT-P-values	CL1 **0.00**	CL2 **0.24**	CL3 0.36	CL4 0.21	CL5 0.05	CL6 0.26	CL7 0.00	CL8 **0.00**	CL9 **0.00**
	**EMA Symptoms**	**CL1**	**CL2**	**CL3**	**CL4**	**CL5**	**CL6**	**CL7**	**CL8**	**CL9**
TEMPORAL LAYER 1	Lack Relaxation	0.82	1.00	0.55	1.00	0.05	0.91	1.00	0.7	0.45
	Loneliness	1.00	0.21	1.00	1.00	0.58	1.00	0.22	0.3	0.08
	Anxiety	0.65	1.00	0.91	1.00	0.39	1.00	1.00	0.9	0.26
	Lack Happiness	0.02	0.20	0.13	0.88	0.27	0.33	0.76	1.0	0.16
	Irritation	0.76	1.00	0.20	1.00	0.48	1.00	1.00	0.2	0.30
	Sensory Issue	0.77	0.90	1.00	1.00	0.05	0.22	0.59	0.0	0.37
	Lack Excitement	1.00	1.00	0.77	0.84	0.00	0.02	0.00	1.0	0.09
	Sadness	0.07	1.00	0.64	1.00	0.23	1.00	1.00	0.0	0.03
	Lack Confidence	0.30	1.00	0.64	1.00	0.35	1.00	1.00	1.0	0.56
	Feeling Rejected	0.62	0.51	1.00	1.00	0.96	0.33	1.00	0.8	0.28
	Feeling Unsafe	0.11	0.07	1.00	1.00	0.28	1.00	0.36	0.8	0.39
	Confusion	0.06	0.11	1.00	1.00	0.35	1.00	0.75	0.3	0.91
	Hallucinations	0.64	0.98	1.00	1.00	0.38	1.00	0.96	0.5	0.36
	Feeling Tired	0.08	0.00	1.00	0.61	0.05	0.91	0.74	0.3	0.14
	Lack Motivation	1.00	1.00	1.00	0.12	1.00	0.08	0.53	1.0	1.00
	Lack Physical Activity	1.00	1.00	1.00	0.38	1.00	0.53	0.46	1.0	0.06
	Finding Activity Difficult	0.75	1.00	1.00	0.89	1.00	0.95	0.38	0.6	1.00
	Lack Enjoying Activity	0.37	1.00	1.00	0.17	1.00	0.47	0.05	0.41	0.05
	Lack Concentration	1.00	1.00	1.00	0.01	0.64	0.12	1.00	1.00	1.00
	Being Alone	1.00	0.40	1.00	1.00	0.37	1.00	0.49	0.20	0.42
	**Symptoms**	**CL1**	**CL2**	**CL3**	**CL4**	**CL5**	**CL6**	**CL7**	**CL8**	**CL9**
TEMPORAL LAYER 2	Lack Relaxation	0.90	1.00	0.10	0.31	1.00	0.00	0.00	0.00	0.99
	Loneliness	0.62	0.63	0.03	0.00	0.10	0.15	0.63	1.00	1.00
	Anxiety	0.44	1.00	0.48	1.00	0.96	0.06	0.06	0.13	1.00
	Lack Happiness	0.35	1.00	0.72	0.03	1.00	0.19	0.36	0.07	1.00
	Irritation	0.78	1.00	1.00	0.08	0.12	0.03	0.00	0.25	1.00
	Sensory Issue	1.00	0.14	0.00	1.00	0.11	0.04	0.00	0.28	1.00
	Lack Excitement	1.00	1.00	0.77	0.51	1.00	0.66	1.00	0.00	0.00
	Sadness	0.03	1.00	0.02	0.01	0.09	0.00	1.00	0.85	1.00
	Lack Confidence	0.49	1.00	0.27	0.06	1.00	0.00	1.00	0.02	1.00
	Feeling Rejected	0.20	0.41	0.07	1.00	1.00	0.02	0.35	0.15	1.00
	Feeling Unsafe	0.26	0.81	1.00	1.00	0.30	0.35	0.00	0.57	1.00
	Confusion	0.12	0.76	1.00	1.00	0.01	0.00	0.00	1.00	1.00
	Hallucinations	1.00	0.99	1.00	1.00	0.00	0.00	0.00	0.89	1.00
	Feeling Tired	1.00	0.86	1.00	0.55	0.06	0.28	0.33	0.62	0.21
	Lack Motivation	1.00	1.00	0.00	0.60	1.00	1.00	1.00	0.21	0.05
	Lack Physical Activity	1.00	1.00	0.00	0.13	1.00	1.00	0.95	1.00	0.00
	Finding Activity Difficult	0.01	1.00	0.00	0.32	1.00	1.00	0.99	0.00	0.00
	Lack Enjoying Activity	0.01	1.00	0.03	0.09	1.00	1.00	0.67	0.00	0.11
	Lack Concentration	1.00	1.00	1.00	0.94	1.00	1.00	1.00	0.00	0.00
	Being Alone	1.00	0.75	0.00	0.00	0.49	1.00	0.82	1.00	1.00

Supplementary Analysis 1: Replication of Behavioral Tractography and Diffusion Analysis in 22q11DS and Typically Developing Sample

Methods

To assess the reproducibility of Behavioral-Tractography and Behavioral Diffusion Analysis (BDA) results, we performed a replication analysis comparing the 22q11DS sample with findings from our previous study on a typically developing population (TD-D) [1]. The goal was to determine whether the composition and average trajectories of Behavioral-Tractography-Bundles observed in the 22q11DS cohort replicate the psychological-contextual interaction patterns previously identified in TD samples. The TD-D sample included 53 typically developing individuals (26 females, mean age = 18.5. SD = 3.7) recruited from the Geneva community and siblings of 22q11DS carriers. All participants had a sufficient command of French, provided written consent and met strict inclusion criteria (excluded individuals with premature birth, a first-degree relative with a developmental disorder (except for a de novo 22q11.2 deletion), or a history of psychiatric, neurological, or learning disorders).
First, we assessed consistency by comparing k-means clustering results of 3D psychological-contextual trajectories in both samples and by correlating the mean trajectory of SP pathways within each Behavioral-Tractography bundle across samples. Finally, we validated Dynamic Behavioral Centrality (DBC) and BDA by correlating their values at individual network nodes between the TD-D and 22q samples.

Results

Replication analyses showed consistency between the 22q11DS and typically developing (TD-D) samples in both Behavioral-Tractography and Behavioral Diffusion Analysis (BDA). As shown in Panel 13, the average 3D coordinates of the nine Behavioral-Tractography-Bundles were correlated across independent samples, with a correlation coefficient of $r=0.87$ ($p<0.001$), indicating reproducibility of bundle composition and trajectories. In Panel 14, BDA trajectories quantified by 8 XY coordinates for each bundle and variable across all 40 network nodes-also showed significant correlation across samples ($r=0.50$, $p<0.001$), supporting the replicability of psychological-contextual interaction patterns. When the analysis was restricted to variables that mediated at least one path in both samples, the correspondence increased ($r=0.80$, $p<0.001$), as indicated by the red regression line. Together, these results indicate that both the composition of Behavioral-Tractography bundles and the underlying BDA trajectories observed in 22q11DS replicate those previously identified in typically developing individuals.

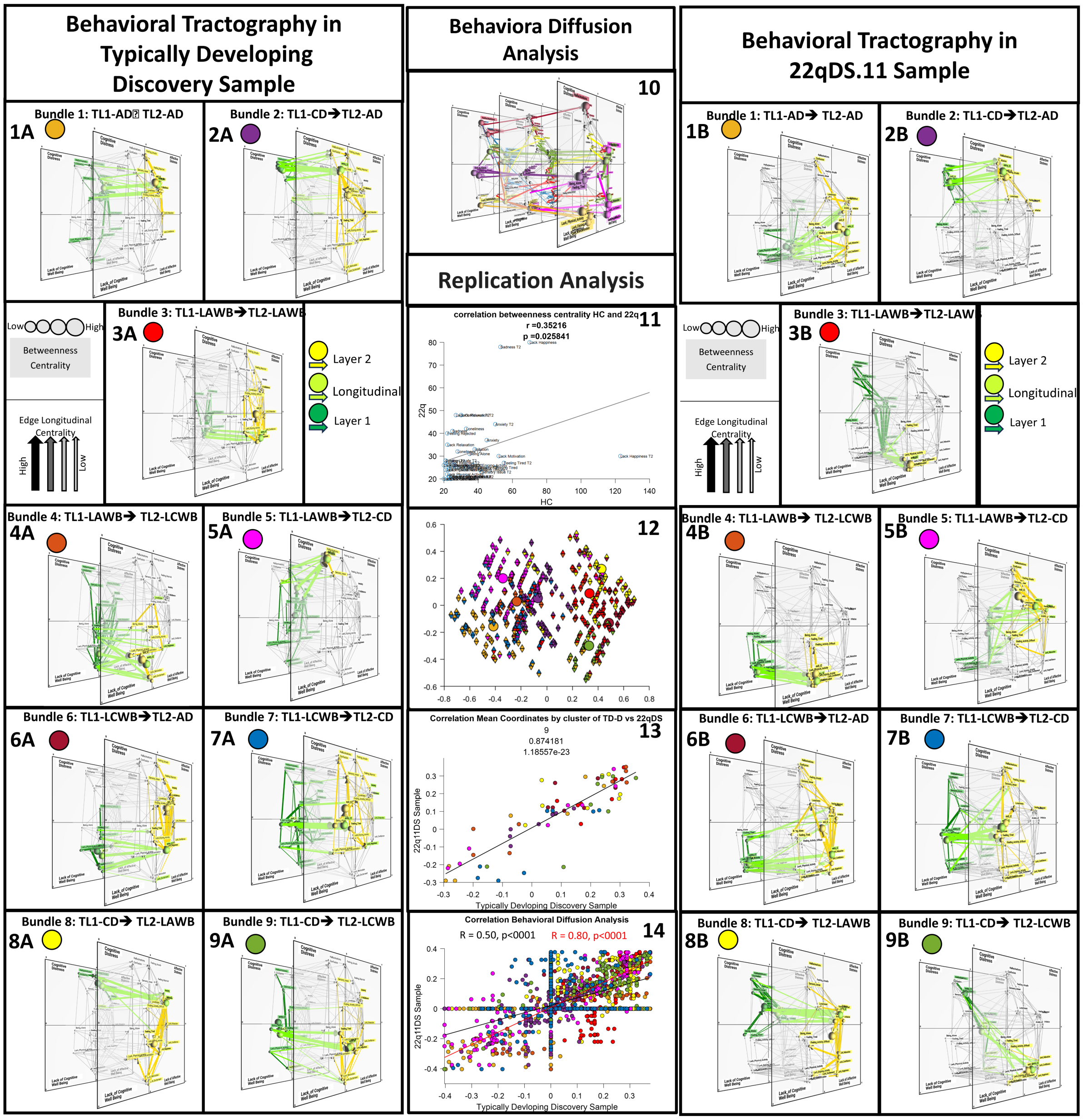

Supplementary figure 1: Replication of Behavioral Tractography and Diffusion Analysis in 22q11DS and Typically Developing Sample. **Panels 1A-1B:** Bundle 1: TL1-AD-->TL2-AD. **Panels 2A-2B:** Bundle 2: TL1-CD-->TL2-AD. **Panels 3A-3B:** Bundle 3: TL1-LAWB-->TL2- LAWB. **Panels 4A-4B:** Bundle 4: TL1- LAWB -->TL2-LCWB. **Panels 5A-5B:** Bundle 5: TL1- LAWB -->TL2-CD. **Panels 6A-6B**: Bundle 6: TL1-LCWB-->TL2-AD. **Panels 7A-7B:** Bundle 7: TL1- LCWB -->TL2-CD. **Panels 8A-8B:** Bundle 8: TL1-CD-->TL2-LAWB. **Panels 9A-9B:** Bundle 9: TL1-CD-->TL2-LCWB. Green to Yellow color coding reflects the dynamic progressing of Shortest Paths from TL1 to TL2 (Start-TL1==>Exit-TL1: Dark Green; Exit-TL1==>Entry-TL2: Light Green; Entry-TL2==>End-TL2: Yellow). **Panels 10:** BDA trajectories of variables that disproportionately mediate PC pathways in each Behavioral-Tractography Bundle in TD-D Sample. Color coding of BDA vectors reflects the Behavioral-Tractography bundle that is significantly mediated by the corresponding variables. Variables that act as gateways for subsequent PC states are represented in TL-1 with their respective BDA trajectories connecting TL-1 and TL-2. Variables that act as funnels for previous PC states are represented in TL-3 with their respective BDA trajectories connecting TL-2 and TL-3. **Panel 11:** Replication analysis of Longitudinal Betweenness Centrality (Longi-BC) across TD-D and 22q samples. **Panel 12:** Replication analysis of Behavioral Tractography across TD-D and 22q samples in terms of composition of Behavioral-Tractography Bundles. Each triangle represents a Psychological-Contextual pathway connecting variables across temporal layers. Pathways are plotted in two-dimensional space derived from Principal-Component-Analysis of their 3D trajectory. PCA was performed on the same data-matrix to which K-means was applied to dissect Behavioral-Tractography bundles. which was composed of a line for each pathway each and 8 columns codifying it's 3-D Trajectory (X-Y-Starting-TL1. X-Y-Exit-TL1. X-Y-Entry-TL2. X-Y-End-TL2). Color of triangles represent the attribution of pathways to one of 9 Behavioral-Tractography-Bundles. Upward-pointing triangles represent composing Behavioral-Tractography Bundles in TD-D sample and downward-pointing triangles in 22q sample. **Panel 13:** Replication of average 3D coordinates of the 9 Behavioral-Tractography-Bundles computed in independent TD-D and 22q sample. Each point represents either the x or y value of one of the 4 coordinates for each bundle. **Panel 14:** Replication analysis of BDA trajectories computed for each variable and each Behavioral-Tractography bundle across TD-D and 22q samples. BDA trajectories are codified by 8 XY coordinates and computed across 9 clusters for all 40 network nodes and yielding a total of 2880 points that are correlated across samples as represented by the black regression line. When a given variable did not mediate any PC-path in a given Behavioral-Tractography bundle the corresponding BDA coordinates were set to 0. We also repeated the BDA replication analysis considering only variables that mediated at least one path in the corresponding Behavioral-Tractography-Bundle in both samples, as represented by the red regression line.

Ema Protocol

General Informations

- 8 notifications per day between 7:30 a.m. and 10:00 p.m. for 6 days
- At least 30 minutes between two notifications
- 15-minute window to respond before the notification disappears
- 3 blocks of questions (mood/symptomatology, context, event)

EMA Items

All EMA items were originally written in French and are presented here translated into English. Items shown in bold indicate those selected for analysis in this paper.

- **Mood/Symptoms**
  - **Right now, I feel relaxed (var = Lack Relaxation)**
  - **Right now, I feel lonely (var = Loneliness)**
  - **Right now, I feel anxious (var = Anxiety)**
  - **Right now, I feel happy, joyful (var = Lack Happiness)**
  - **Right now, I feel irritated, angry (var = Irritation)**
  - **Right now, I feel bothered by sensory stimulation (var = Sensory Issue)**
  - **Right now, I feel excited (var = Lack Excitement)**
  - **Right now, I feel sad (var = Sadness)**
  - **Right now, I feel confident (var = Lack Confidence)**
  - **Right now, I feel like others don't like me (var = Feeling Rejected)**
  - **Right now, I feel like I have to stay on guard, that I'm not safe (var = Feeling Unsafe)**
  - **Right now, I feel like my imagination is blending with reality (var = Confusion)**
  - **Right now, I feel like I'm hearing or seeing things that others don't perceive (var = Hallucinations)**
  - **Right now, I feel tired (var = Feeling Tired)**
  - **Right now, I feel like doing many things, I feel motivated (var = Lack Motivation)**
  - **Since the last beep, I have been physically active (var = Lack Physical Activity)**

- **Contexte**
  - What are you doing? (just before the beep) (var = Cur_activ)
  - **This activity is difficult (var = Finding Activity Difficult)**
  - **I am enjoying this activity (var = Lack Enjoying Activity)**
  - **I am focused on this activity (var = Lack Concentration)**
  - Where are you? (juste avant le beep) (var = Location)
  - **Are you alone? (var = Being Alone)**
  - Yes (=1)
    - I would prefer to be with other people (var = Pref_other)
    - I like being alone (var = Likealone)
    - I feel excluded, rejected (var = Feel_exclu)
  - No (=0)
    - Who are you with?
    - I would prefer to be alone (var = Pref_alone)
    - This company is pleasant (var = likecomp)

    - We are doing something together (var = interact)
    - I feel judged by this/these person(s) (var = feel_judged)
    - I am nervous in the presence of this/these person(s) (var = feel_nerv)
- Evénement
    - Now think about the most important event that has happened since the last beep
    - This event was pleasant
    - This event was stressful
    - This event was important
    - Were you alone during this event?
    - Now think about the most important event that will happen in the next hour
    - I am looking forward to this event
    - In which category does this event fall?
    - Will you be alone during this event?

Response scale:

1 (not at all), 2 (very slightly), 3 (slightly), 4 (moderately), 5 (strongly), 6 (very strongly), 7 (extremely)

Context: activity:
10 (work/school), 20 (household tasks), 30 (eating or drinking), 40 (personal care), 50 (rest/relaxation), 60 (social contact), 65 (online social contact), 70 (sports), 75 (TV, internet), 79 (other leisure), 89 (something else), 0 (nothing)

Context: location:
10 (home), 20 (school/work), 30 (at a friend's place), 40 (at a family member's place), 50 (hospital or care facility), 60 (public place), 70 (in transit), 89 (elsewhere)

Context: with whom:
10 (person living with me), 20 (person not living with me), 30 (boyfriend/girlfriend, spouse), 40 (friend), 50 (classmate), 60 (healthcare professional), 70 (acquaintance), 80 (pet), 89 (stranger), 99 (no one else)

Event:
0 (nothing), 10 (physical activity), 20 (work/school), 30 (leisure), 40 (sleeping), 50 (eating/drinking), 60 (medical activity), 89 (other)

1. Sandini, C., et al., *More than the sum of their parts: A Behavioral Tractography analysis of distress wellbeing and context reveals dynamic signatures of psychosis and autism.* medRxiv, 2025: p. 2025.07.09.25330999.
